# A nuclear ERK–ATF5 axis coordinates mitochondrial metabolism and thermogenic gene expression in brown adipocytes

**DOI:** 10.1530/JOE-26-0101

**Published:** 2026-10-19

**Authors:** Hyo Ju Jang, Yeongjun Suh, Jingwen Tian, Ho Yeop Lee, Ji Sun Moon, Ha Thi Nga, Thi Linh Nguyen, Alfin Mohammad Abdillah, Gyu Min Lim, Dong Wook Choi, Chuna Kim, Yonghoon Kwon, Hyon-Seung Yi

**Affiliations:** ^1^ Laboratory of Endocrinology and Immune System, Chungnam National University School of Medicine, Daejeon, Republic of Korea; ^2^ Department of Medical Science, Chungnam National University School of Medicine, Daejeon, Republic of Korea; ^3^ Department of Life Sciences, Gwangju Institute of Science and Technology, Gwangju, Republic of Korea; ^4^ Integrated Institute of Biomedical Research, Gwangju Institute of Science and Technology, Gwangju, Republic of Korea; ^5^ Department of Orthopaedic Surgery, The Sixth Affiliated Hospital of Sun Yat-sen University, Guangzhou, Guangdong, P.R. China; ^6^ Department of Biotechnology, College of Life Sciences and Biotechnology, Korea University, Seoul, Republic of Korea; ^7^ Aging Convergence Research Center, Korea Research Institute of Bioscience and Biotechnology (KRIBB), Daejeon, Republic of Korea; ^8^ Department of Bioinformatics, KRIBB School of Bioscience, Korea University of Science and Technology, Daejeon, Republic of Korea; ^9^ Department of Internal Medicine, Chungnam National University School of Medicine, Daejeon, Republic of Korea

**Keywords:** brown adipocytes, ATF5, thermogenesis, PKA–ERK, mitochondrial metabolism

## Abstract

**Graphical abstract:**

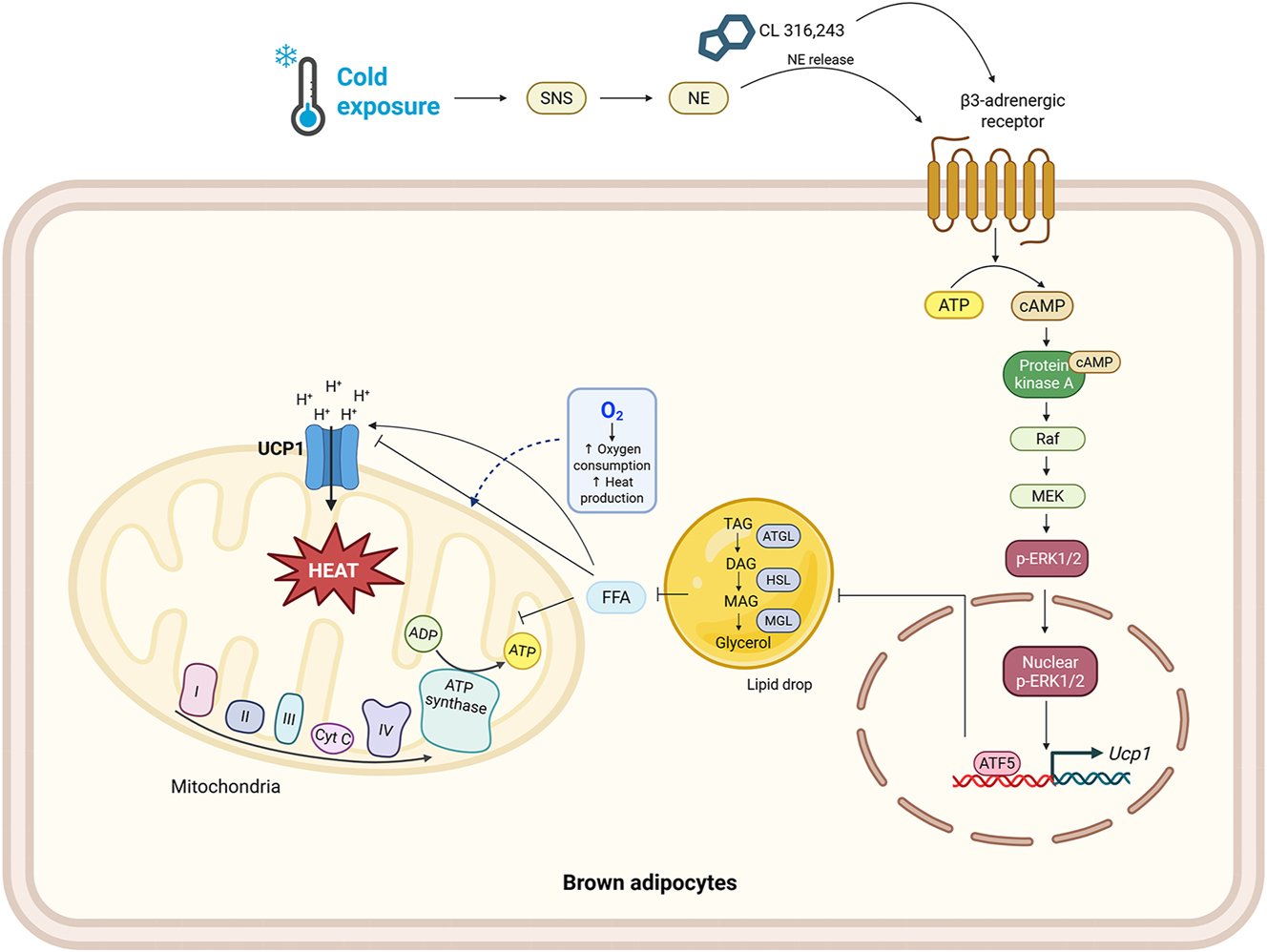

**Abstract:**

Obesity and diabetes arise from disrupted energy homeostasis, underscoring the need to identify mechanisms that enhance energy expenditure. Brown adipose tissue dissipates energy through mitochondria-dependent non-shivering thermogenesis; however, the transcriptional regulators coordinating this process remain incompletely defined. Here, we investigated the role of activating transcription factor 5 in thermogenic programming and mitochondrial metabolism in brown adipocytes using *in vivo* and *in vitro* models. Cold exposure and β3-adrenergic stimulation induced activating transcription factor 5 expression in brown adipose tissue and differentiated adipocytes, accompanied by activation of thermogenic gene programs. Mechanistically, activating transcription factor 5 functioned downstream of a nuclear protein kinase A–extracellular signal-regulated kinase pathway, whose nuclear activation was required for its induction and for upregulation of uncoupling protein 1. Loss of activating transcription factor 5 impaired thermogenic gene expression, reduced mitochondrial stress response and lipolytic programs, diminished mitochondrial oxidative capacity, and suppressed fatty acid oxidation, including reduced incorporation of fatty acid–derived carbons into the tricarboxylic acid cycle. Transcriptomic analysis revealed coordinated repression of pathways related to fatty acid oxidation, oxidative phosphorylation, and thermogenesis in activating transcription factor 5–deficient adipocytes. Live-cell imaging further confirmed that β3-adrenergic stimulation selectively enhanced nuclear kinase activity, establishing a nuclear-localized signaling axis. Together, these findings identify activating transcription factor 5 as a key transcriptional regulator linking mitochondrial metabolism and thermogenic gene expression, supporting its potential as a therapeutic target for enhancing energy expenditure in metabolic disease.

## Introduction

Obesity arises from a chronic imbalance between energy intake and expenditure, underscoring the importance of identifying pathways that enhance energy dissipation (1). Brown adipose tissue (BAT) increases whole-body energy expenditure through mitochondria-dependent non-shivering thermogenesis and improves systemic metabolic homeostasis (2, 3). Thermogenic activation is initiated by cold-induced sympathetic signaling that engages β3-adrenergic receptors (β3-ARs) and activates protein kinase A (PKA) (4, 5). PKA-driven lipolysis liberates fatty acids that both fuel oxidative phosphorylation and activate uncoupling protein 1 (UCP1), the central effector of heat production (6, 7, 8). Although β3-adrenergic signaling has been extensively characterized, the nuclear transcriptional programs that couple lipolytic cues to mitochondrial adaptive responses during thermogenesis remain incompletely defined (4, 5).

Activating transcription factor 5 (ATF5) is a stress-responsive transcription factor that regulates mitochondrial proteostasis through the mitochondrial unfolded protein response (UPRmt) (9, 10). ATF5 is induced by mitochondrial perturbations and coordinates adaptive remodeling of oxidative metabolism (11, 12). However, whether ATF5 participates in brown adipocyte thermogenic activation has not been investigated. Given that sustained thermogenesis imposes substantial mitochondrial and metabolic stress, we hypothesized that ATF5 may function as a transcriptional integrator linking lipolytic signaling to mitochondrial adaptation in BAT (13, 14).

Here, we demonstrate that cold exposure and β3-adrenergic stimulation activate a nuclear-localized ERK1/2–ATF5 signaling axis in brown adipocytes. Nuclear ERK activation is required for ATF5 induction and subsequent upregulation of UCP1-dependent thermogenic programs. Loss of *Atf5* impairs fatty acid oxidation, oxidative phosphorylation, lipolytic gene expression, and UPRmt signaling, revealing its essential role in coordinating metabolic and stress-adaptive pathways. Together, these findings identify a previously unrecognized nuclear PKA–ERK–ATF5 axis that integrates lipid catabolism with mitochondrial stress adaptation to drive thermogenic activation, positioning ATF5 as a central regulator of BAT metabolism and a potential therapeutic target for metabolic disease.

## Materials and methods

### Animal experiment

Male C57BL/6 wild-type mice (8 weeks old; DooYeol Biotech, South Korea) were maintained in a specific pathogen-free facility under a 12 h light:12 h darkness cycle with *ad libitum* access to food and water. Mice were individually housed and exposed to 8°C for 48 h or 14°C for 5 days. For transcriptomic analysis, brown adipocyte-specific *Atf5* knockout mice (Ucp1-Cre; Atf5^fl/fl^) and littermate control mice were maintained under basal conditions without cold exposure, and BAT tissues were collected for RNA sequencing. All animal procedures, including cold exposure studies and experiments using brown adipocyte-specific *Atf5* knockout mice, were approved by the Institutional Animal Care and Use Committee of Chungnam National University Hospital (CNUH-2023-IA0019).

### Cell culture and differentiation

WT-1 mouse brown preadipocytes (MilliporeSigma, USA; SCC255) were cultured in DMEM supplemented with 10% fetal bovine serum (FBS) and 1% penicillin–streptomycin in a 5% CO_2_ incubator at 37°C. Differentiation was induced by culturing the cells in DMEM containing 10% FBS, 20 nM insulin, 1 nM triiodo-L-thyronine (T3), 0.125 mM indomethacin, 5 μM dexamethasone, and 0.5 mM IBMX for 2 days. Cells were then maintained in DMEM containing 10% FBS, insulin (20 nM), and T3 (1 nM) for 7 additional days. For pharmacological inhibition, cells were pretreated with KT5720 (1 μM) or LY3214996 (1 μM) for 1 h and then treated with vehicle or CL-316,243 (1 μM) for 30 min (phosphorylation) or 6 h (ATF5/UCP1), with the inhibitor maintained. Basal-condition samples were harvested 15–60 min after treatment with the inhibitor alone. Inhibitor-free samples were treated with CL-316,243 (1 μM) for 6 h.

### Transfection

For imaging and proximity ligation assays, WT-1 cells were plated onto sterile poly-D-lysine–coated 35-mm glass-bottom dishes or glass coverslips and grown to 50–70% confluence before transfection with Lipofectamine 2000 (Invitrogen, Thermo Fisher Scientific, USA) according to the manufacturer’s instructions. Cells were maintained for 16–24 h before imaging. For siRNA-mediated knockdown experiments, differentiated brown adipocytes were transfected with siAtf5 using Lipofectamine RNAiMAX (Invitrogen). Cells (2.5 × 10^5^ per well) were transfected with 100 or 200 nM siAtf5 complexes prepared in Opti-MEM (Thermo Fisher Scientific) and incubated for 24 h at 37°C.

### RNA extraction and real-time PCR analysis

Total RNA was isolated from BAT or differentiated adipocytes using TRIzol Reagent (Thermo Fisher Scientific). First-strand cDNA was synthesized from 1 μg total RNA using M-MLV Reverse Transcriptase and oligo(dT) primers (Invitrogen). Quantitative real-time PCR was performed using SYBR Green Master Mix (Applied Biosystems, Thermo Fisher Scientific) on a 7500 Fast Real-Time PCR System. Reactions were performed in triplicate. Gene expression was normalized to 18S rRNA, which was confirmed to be stable across experimental conditions, and quantified using the ΔΔCt method. All primer sequences used for qPCR analyses are listed in Supplementary Table 1 (see the section on Supplementary materials given at the end of the article).

### Western blot analysis

Cells or tissues were lysed in lysis buffer containing Tris–HCl, NaCl, EDTA, and Triton X-100 supplemented with protease and phosphatase inhibitors. Proteins (20 μg) were separated by SDS–PAGE and transferred to nitrocellulose membranes. Membranes were blocked and incubated overnight at 4°C with primary antibodies, followed by incubation with HRP-conjugated secondary antibodies. Signals were detected using enhanced chemiluminescence (ECL). Antibodies used in this study are listed in Supplementary Table 2.

### Nuclear and cytoplasmic protein extraction

Cytoplasmic and nuclear fractions were prepared using the NE-PER Nuclear and Cytoplasmic Extraction Kit (Thermo Fisher Scientific) according to the manufacturer’s instructions, with minor modifications for tissue homogenization. Fraction purity was confirmed by immunoblotting for Lamin B1 (nuclear marker) and GAPDH (cytoplasmic marker).

### Seahorse metabolic analysis

Mitochondrial respiration and glycolytic activity were assessed by measuring the oxygen consumption rate (OCR) and extracellular acidification rate (ECAR) using an XF96 Extracellular Flux Analyzer (Agilent Technologies, USA). Differentiated adipocytes were seeded at 1 × 10^4^ cells per well. After equilibration in Seahorse XF base medium, oligomycin (2 μg/mL), FCCP (10 μM), and rotenone/antimycin A (1 μM each) were sequentially injected. Data were normalized to protein content.

### Fluorescence imaging and FRET biosensor analysis

Cells were washed twice with Hank’s balanced salt solution (HBSS) and incubated at 37°C for 30 min before imaging. Cells were subsequently imaged in HBSS in the dark at 37°C. Epifluorescence imaging was performed on a Leica Thunder microscope (Leica, Germany) equipped with a 40×/1.3 NA objective and a Leica K8 sCMOS camera (Leica) controlled by LAS X software (Leica). The following excitation/emission filter combinations were used: CFP – EM473/22, DFT51010 filter cube (11525418, Leica), 459-nm dichroic mirror, EX436/28; YFP – EM539/24, DFT51010 filter cube (11525418, Leica), 523-nm dichroic mirror, EX406/21; CYFRET – EM539/24, DFT51010 filter cube (11525418, Leica), 459-nm dichroic mirror, EM473/22. Exposure times were 50 ms for the CYFRET channel and 250 ms for the CFP channel, and images were acquired every 30 s. Fluorescence images were background-corrected by subtracting the background fluorescence intensity of a cell-free region from the emission intensities of biosensor-expressing cells. Yellow/cyan emission ratios for EKAR4 were then calculated at each time point. Time-course data were normalized by dividing the ratio at each time point by the basal value at time zero (R/R_0_), which was defined as the last time point immediately preceding drug treatment. Maximum ratio changes (ΔR/R_0_) were calculated as (Rmax − R_0_)/R_0_, where Rmax is the maximum ratio value recorded after drug treatment.

### Citrate flux measurement using GC–MS

For U-^13^C-palmitate tracing, cells were cultured with 10 mM U-^13^C-palmitate conjugated to BSA for 1 h. Metabolites were extracted using 80% methanol containing 100 μM DL-norvaline (N7502, Sigma-Aldrich, USA) as an internal standard. Cells were lysed by sonication (BioRuptor, Diagenode, Belgium) using 30 s on/off cycles for 10 min at 4°C and centrifuged at 15,100 *g* for 15 min at 4°C. The supernatant was transferred to a new tube and dried in a vacuum centrifuge for 4 h at 4°C. Samples were derivatized with 30 μL methoxyamine hydrochloride solution (10 mg/mL in pyridine; Sigma-Aldrich) at 37°C for 30 min, followed by the addition of 70 μL N-tert-butyldimethylsilyl-N-methyltrifluoroacetamide (Sigma-Aldrich) and incubation at 70°C for 1 h. GC–MS analysis was performed using an Agilent 8890 GC coupled to a 5977B mass selective detector (Agilent Technologies). Derivatized samples (1 μL) were injected in splitless mode using an Agilent 7693A autosampler and separated on an Agilent J&W HP-5 ms capillary column with helium as the carrier gas (1.5 mL/min). The oven temperature was programmed from 6 to 320°C at 10°C/min. Mass detection was performed in electron ionization mode (70 eV). Data acquisition and isotopologue quantification were performed using MassHunter software (Agilent Technologies). Citrate (*M* = 459) isotopologues were determined by adding the number of incorporated ^13^C atoms to the base molecular weight.

### RNA sequencing analysis

Total RNA was extracted as described above. RNA concentration and integrity were assessed using Qubit fluorometry and a Bioanalyzer or TapeStation, and only samples with an RNA integrity number (RIN) ≥ 7.0 were used. Stranded RNA-seq libraries with rRNA depletion were prepared using the Illumina Stranded Total RNA Prep, Ligation with Ribo-Zero Plus Kit on a Hamilton NGS STAR robot. Libraries were quality-checked by qPCR and Bioanalyzer and sequenced on an Illumina NovaSeq 6000 platform (2 × 100 bp paired-end), targeting a minimum of 25 million reads per sample. Raw BCL files were demultiplexed and converted to FASTQ format using DRAGEN (v4.4.4). Reads were aligned to the mouse reference genome (mm10) using STAR (v2.7.10a), and gene-level counts were generated using featureCounts (v2.0.3). For BAT RNA-seq analysis, two biological replicates per group (Ucp1-Cre; Atf5^fl/fl^ and littermate controls) were used because of the limited availability of BAT-specific *Atf5* knockout mice. Therefore, transcriptomic analyses were considered exploratory and supportive. Differential gene expression analysis was performed using DESeq2 (v1.38.3) with a false discovery rate (FDR) threshold of <0.05. Principal component analysis (PCA) was conducted on variance-stabilized counts generated by DESeq2 to assess global transcriptional differences between groups. Hierarchical clustering and heatmaps were generated based on normalized expression values. All analyses were performed in R (v4.3.0). RNA-seq data have been deposited in the Gene Expression Omnibus (GEO) under accession number GSE315093.

### Statistical analysis

Statistical analyses were performed using GraphPad Prism (version 9; GraphPad Software, USA). Data are presented as mean ± SD unless otherwise indicated. Comparisons between two groups were performed using an unpaired two-tailed Student’s *t*-test, and multiple-group comparisons were performed using one-way ANOVA followed by Dunnett’s multiple comparisons test. Biosensor imaging data are presented as mean ± SEM. A *P*-value <0.05 was considered statistically significant.

## Results

### Cold exposure induces ATF5 and thermogenic gene expression in brown adipose tissue

To determine whether *Atf5* is regulated by cold exposure, we first analyzed publicly available transcriptomic data (GSE147392) from wild-type mice maintained under thermoneutral (30°C) or cold (5°C) conditions (15). Cold exposure increased *Atf5* and *Ucp1* mRNA expression in brown adipose tissue (BAT) (Fig. 1A). Heatmap analysis further revealed coordinated upregulation of genes involved in lipid metabolism, β-oxidation, and mitochondrial pathways in cold-exposed BAT compared with thermoneutral controls (Fig. 1B).

**Figure 1:**
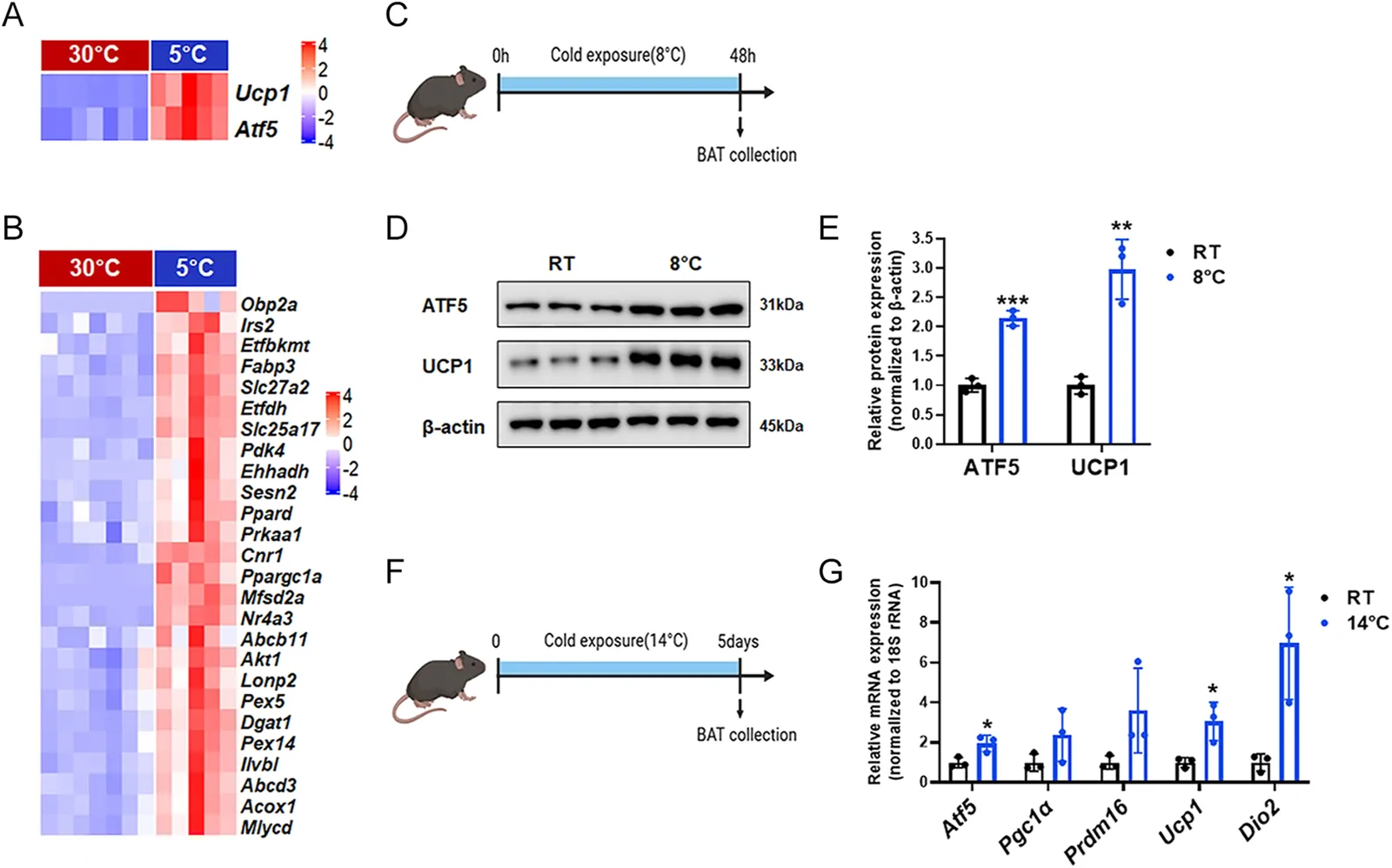
Cold exposure induces ATF5 and thermogenic gene expression in brown adipose tissue *in vivo*. (A) Heatmap showing the expression of *Ucp1* and *Atf5* in BAT from wild-type mice housed at 30°C or 5°C (GSE147392) (15). (B) Heatmap of lipid metabolism–associated genes in BAT from mice housed at 30°C or 5°C. (C) Schematic of the acute cold exposure protocol (8°C for 48 h). (D) Representative immunoblots of ATF5 and UCP1 in BAT from mice housed at room temperature (RT) or 8°C for 48 h. β-actin served as a loading control (*n* = 3 mice per group). (E) Quantification of ATF5 and UCP1 protein levels normalized to β-actin (*n* = 3 mice per group). (F) Schematic of the chronic cold exposure protocol (14°C for 5 days). (G) Relative mRNA expression of thermogenic genes (*Atf5*, *Pgc1α*, *Prdm16*, *Ucp1*, and *Dio2*) in BAT from mice housed at RT or 14°C for 5 days. Gene expression was determined by qRT-PCR, normalized to 18S rRNA, and expressed relative to RT control (*n* = 3 mice per group). Data are presented as mean ± SD from *n* = 3 independent experiments unless otherwise indicated. Statistical significance was determined using an unpaired two-tailed Student’s *t*-test or one-way ANOVA followed by Dunnett’s multiple comparisons test, as indicated. *P < 0.05, **P < 0.01 versus RT control. A full-color version of this figure is available at https://doi.org/10.1530/JOE-26-0101.

To validate these findings *in vivo*, wild-type mice were subjected to acute cold exposure at 8°C for 48 h (Fig. 1C). Immunoblot analysis demonstrated increased ATF5 and UCP1 protein levels in BAT relative to room temperature (RT) controls (Fig. 1D), and densitometric quantification confirmed the induction of both proteins (Fig. 1E).

To assess transcriptional responses under sustained cold conditions, mice were housed at 14°C for 5 days (Fig. 1F). qRT-PCR analysis showed elevated expression of *Atf5*, as well as key thermogenic regulators, including *Pgc1α*, *Prdm16*, *Ucp1*, and *Dio2*, in BAT from cold-exposed mice (Fig. 1G). These experiments were performed under distinct cold paradigms (acute exposure at 8°C and sustained exposure at 14°C) to model varying degrees of physiological cold stress.

Consistent with its potential role in brown adipocyte biology, ATF5 expression progressively increased during brown adipocyte differentiation (Supplementary Fig. 1).

Collectively, these findings indicate that ATF5 is induced by cold exposure and is associated with the thermogenic transcriptional program in BAT.

Given that β3-adrenergic signaling is a primary mediator of cold-induced thermogenesis, we next examined whether ATF5 expression is regulated by β3-adrenergic stimulation in brown adipocytes (4, 5). Additional analysis of the public dataset GSE147392 revealed that several members of the ATF family exhibited altered expression in response to cold exposure (Supplementary Fig. 4E). Among these factors, ATF5 was prioritized for further investigation because of its robust induction by cold exposure and its established role in mitochondrial stress adaptation. Collectively, these observations prompted us to further examine whether ATF5 is regulated by β3-adrenergic signaling in brown adipocytes.

### β3-adrenergic signaling induces ATF5 and regulates thermogenic gene expression in brown adipocytes

To examine whether β3-adrenergic signaling regulates *Atf5* expression, differentiated brown adipocytes were treated with the β3-adrenergic receptor agonist CL-316,243 (CL). CL treatment increased ATF5 and UCP1 protein levels compared with vehicle-treated controls (Fig. 2A and B). In parallel, mRNA analysis revealed elevated expression of *Atf5* and *Ucp1* following CL treatment (Fig. 2C), indicating that ATF5 expression is responsive to β3-adrenergic activation.

**Figure 2:**
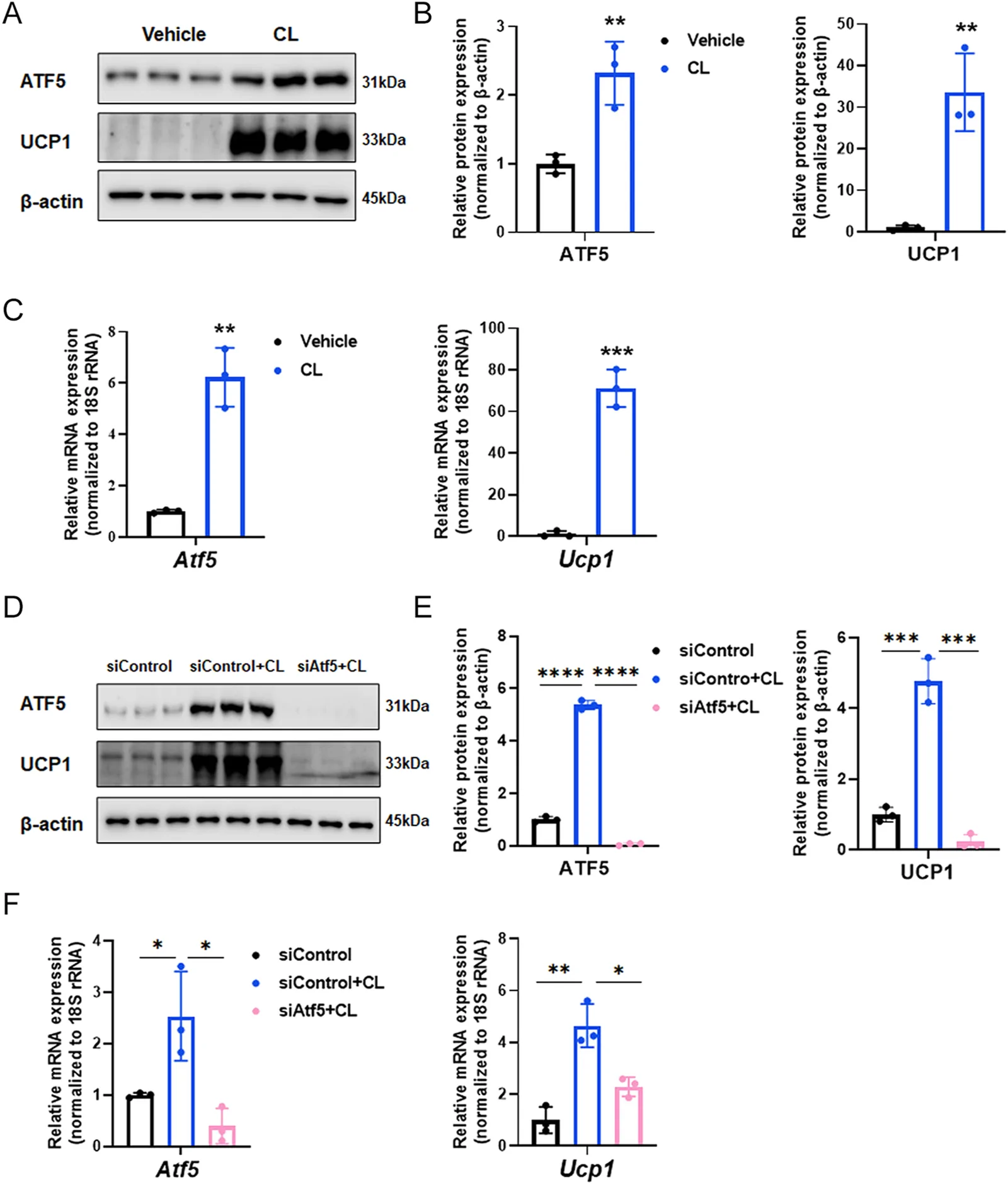
β3-adrenergic stimulation induces ATF5 and promotes thermogenic gene expression in brown adipocytes *in vitro*. (A) Representative immunoblots of ATF5 and UCP1 in differentiated brown adipocytes treated with vehicle or CL-316,243 (1 μM, 6 h). β-actin served as a loading control (*n* = 3). (B) Quantification of ATF5 and UCP1 protein levels normalized to β-actin (*n* = 3). (C) Relative mRNA expression of *Atf5* and *Ucp1* following CL treatment (1 μM, 6 h). Gene expression was determined by qRT-PCR, normalized to 18S rRNA, and expressed relative to vehicle (*n* = 3). (D) Representative immunoblots of ATF5 and UCP1 in differentiated brown adipocytes transfected with siControl or siAtf5 and subsequently treated with CL-316,243 (1 μM, 6 h). β-actin served as a loading control (*n* = 3). (E) Quantification of ATF5 and UCP1 protein levels normalized to β-actin (*n* = 3). (F) Effect of *Atf5* knockdown on CL-induced thermogenic gene expression. Differentiated brown adipocytes were transfected with siControl or siAtf5 and treated with CL (1 μM, 6 h). mRNA levels were normalized to 18S rRNA and expressed relative to siControl (*n* = 3). Data are presented as mean ± SD from *n* = 3 independent experiments performed in cultured brown adipocytes. Statistical significance was determined using an unpaired two-tailed Student’s *t*-test or one-way ANOVA followed by Dunnett’s multiple comparisons test, as indicated. *P < 0.05, **P < 0.01 versus siControl or vehicle as indicated. A full-color version of this figure is available at https://doi.org/10.1530/JOE-26-0101.

To determine whether ATF5 contributes to adrenergic induction of thermogenic genes, Atf5 was silenced using siRNA before CL stimulation. Immunoblot analysis demonstrated that siAtf5 effectively reduced ATF5 protein expression and markedly attenuated CL-induced UCP1 protein expression (Fig. 2D and E). Consistent with these findings, knockdown of *Atf5* also significantly reduced CL-induced Ucp1 mRNA expression (Fig. 2F). These results suggest that ATF5 participates in the transcriptional activation of thermogenic genes downstream of β3-adrenergic receptor signaling.

Collectively, these findings identify ATF5 as a β3-adrenergic-responsive factor that contributes to UCP1 induction in brown adipocytes.

### ATF5 is positioned downstream of the PKA–ERK signaling axis in brown adipocytes

Activation of β3-adrenergic receptors stimulates the PKA signaling cascade, which drives thermogenic activation in BAT (4, 5). To determine whether ATF5 is regulated through this pathway, differentiated brown adipocytes were treated with the PKA inhibitor KT5720 (1 μM) for 15, 30, or 60 min. Inhibition of PKA activity was confirmed by a time-dependent reduction in CREB phosphorylation, a canonical downstream target of PKA. Notably, phosphorylated ERK (p-ERK) levels decreased as early as 15 min after KT5720 treatment, indicating that ERK activation occurs downstream of PKA signaling. Consistent with these changes, ATF5 and UCP1 protein levels were reduced after 60 min of PKA inhibition (Fig. 3A and B), suggesting that ATF5 expression depends on PKA activity during thermogenic induction.

**Figure 3:**
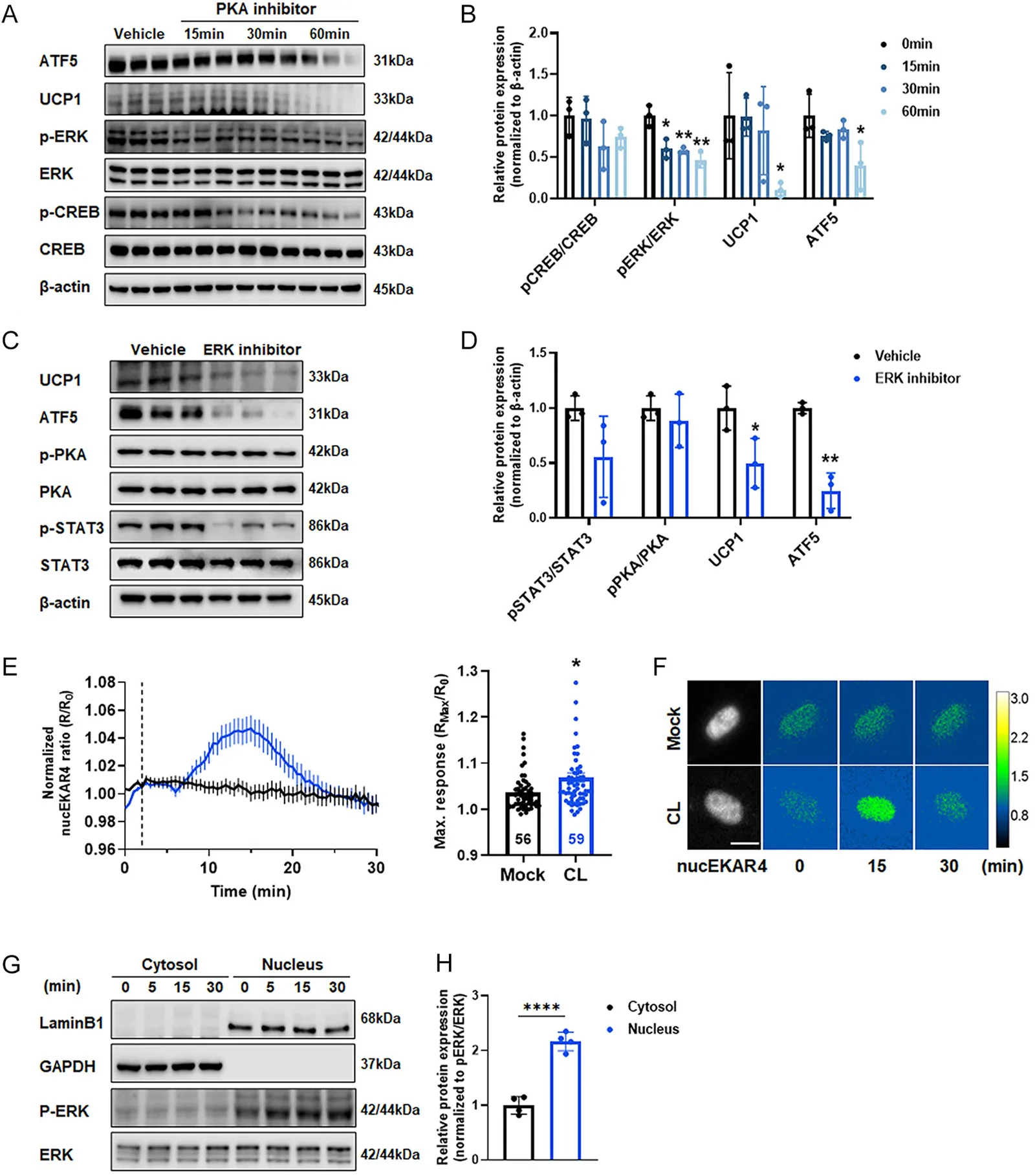
ATF5 expression and thermogenic activation are regulated through the PKA–ERK signaling pathway *in vitro* and *in vivo*. (A) Representative immunoblots showing ERK and CREB phosphorylation and ATF5 and UCP1 protein levels in differentiated brown adipocytes treated with the PKA inhibitor KT5720 (1 μM) for 15, 30, or 60 min. β-actin served as a loading control (*n* = 3). (B) Quantification of p-ERK/ERK, p-CREB/CREB, UCP1, and ATF5 protein levels. Phosphorylated protein levels were normalized to total protein levels, and total protein levels were normalized to β-actin and expressed relative to vehicle (*n* = 3). (C) Representative immunoblots showing the effects of the ERK inhibitor LY3214996 (1 μM, 1 h) on ATF5 and UCP1 protein levels, and PKA and STAT3 phosphorylation. (D) Quantification of p-STAT3/STAT3, p-PKA/PKA, UCP1, and ATF5 protein levels normalized as described above (*n* = 3). (E) Representative Y/C emission ratio time courses of nuclear ERK activity monitored using the nucEKAR4 biosensor in WT-1 brown adipocytes (left). Cells were treated with vehicle (HBSS; black) or CL-316,243 (10 μM; blue) at the time indicated by the arrowhead and dashed line. Quantification of maximal nuclear ERK responses measured by nucEKAR4 in WT-1 cells is shown on the right (*n* = 56 for Mock; *n* = 59 for CL-316,243 from three independent experiments). Data are presented as mean ± SEM. Statistical significance was determined using an unpaired two-tailed Student’s *t*-test. The exact *P* value is indicated in the plot. (F) Representative fluorescence images of differentiated WT-1 brown adipocytes expressing nucEKAR4 (left) and corresponding pseudocolored Y/C emission ratio images at the indicated time points following CL-316,243 stimulation (10 μM). Warmer colors indicate higher nuclear ERK activity. (G) Immunoblot analysis of cytosolic and nuclear fractions from BAT of mice exposed to cold (8°C) for the indicated times, showing p-ERK and total ERK distribution. GAPDH and Lamin B1 served as cytosolic and nuclear markers, respectively (*n* = 3). (H) Quantification of p-ERK/ERK ratios in cytosolic and nuclear fractions (*n* = 3). Data are presented as mean ± SD unless otherwise indicated. Statistical significance was determined using an unpaired two-tailed Student’s *t*-test or one-way ANOVA followed by Dunnett’s multiple comparisons test, as indicated. *P < 0.05, **P < 0.01. Panels A–F represent experiments performed in cultured brown adipocytes (*in vitro*), whereas panels G and H represent experiments performed in BAT from cold-exposed mice (*in vivo*). A full-color version of this figure is available at https://doi.org/10.1530/JOE-26-0101.

Because ERK phosphorylation was diminished upon PKA inhibition, we next examined whether ERK directly regulates ATF5 and thermogenic gene expression. Treatment with the ERK inhibitor LY3214996 (1 μM for 1 h) did not alter PKA phosphorylation but significantly reduced ATF5 and UCP1 protein levels (Fig. 3C and D), indicating that ERK functions downstream of PKA to regulate ATF5 expression.

β3-adrenergic signaling has been reported to regulate target gene expression through the accumulation of nuclear ERK activity (16, 17). To determine whether β3-AR stimulation induces nuclear ERK activation in brown adipocytes, we employed a nucleus-targeted FRET-based ERK activity reporter (nucEKAR4) in differentiated WT-1 brown adipocytes. CL-316,243 stimulation elicited a rapid and sustained increase in the nuclear Y/C emission ratio (Fig. 3E, left), indicating enhanced nuclear ERK activity. Quantitative analysis confirmed a significant increase in maximal nuclear ERK responses compared with vehicle-treated controls (Fig. 3E, right). Representative nucEKAR4 fluorescence images and corresponding pseudocolored Y/C emission ratio images further visualized the temporal increase in nuclear ERK activity following CL-316,243 stimulation (Fig. 3F).

Importantly, similar activation of nuclear ERK was observed in undifferentiated brown adipocytes expressing nucEKAR4, as demonstrated by elevated maximal Y/C emission ratios at the single-cell level (Supplementary Fig. 2). These findings indicate that β3-adrenergic stimulation robustly induces nuclear ERK activity irrespective of differentiation status.

To further validate these observations *in vivo*, cytosolic and nuclear fractions were isolated from BAT of mice exposed to 8°C. Cold exposure increased nuclear p-ERK abundance without affecting total ERK levels, resulting in a marked elevation of the nuclear p-ERK/ERK ratio (Fig. 3G and H).

Collectively, these data position ATF5 within the canonical β3-adrenergic–PKA–ERK signaling cascade and support a model in which β3-adrenergic stimulation promotes the accumulation of nuclear ERK activity to facilitate ATF5-associated thermogenic gene transcription in brown adipocytes.

### The PKA–ERK axis is required for adrenergically induced ATF5 and UCP1 expression

The experiments described above demonstrated that pharmacological inhibition of PKA or ERK reduced ATF5 and UCP1 expression under basal conditions. To determine whether this signaling axis is similarly required during active adrenergic stimulation, we treated differentiated brown adipocytes with KT5720 or LY3214996 before CL-316,243 stimulation. CL-316,243 induced phosphorylation of ERK and CREB within 30 min, and this induction was markedly attenuated by pretreatment with KT5720 (Fig. 4C and D). Consistent with the reduction in nuclear ERK signaling, CL-316,243-induced ATF5 and UCP1 protein expression at 6 h was similarly suppressed by KT5720 pretreatment (Fig. 4A and B), indicating that PKA activity is required for adrenergically induced ATF5 and UCP1 expression and not merely for their basal turnover.

**Figure 4:**
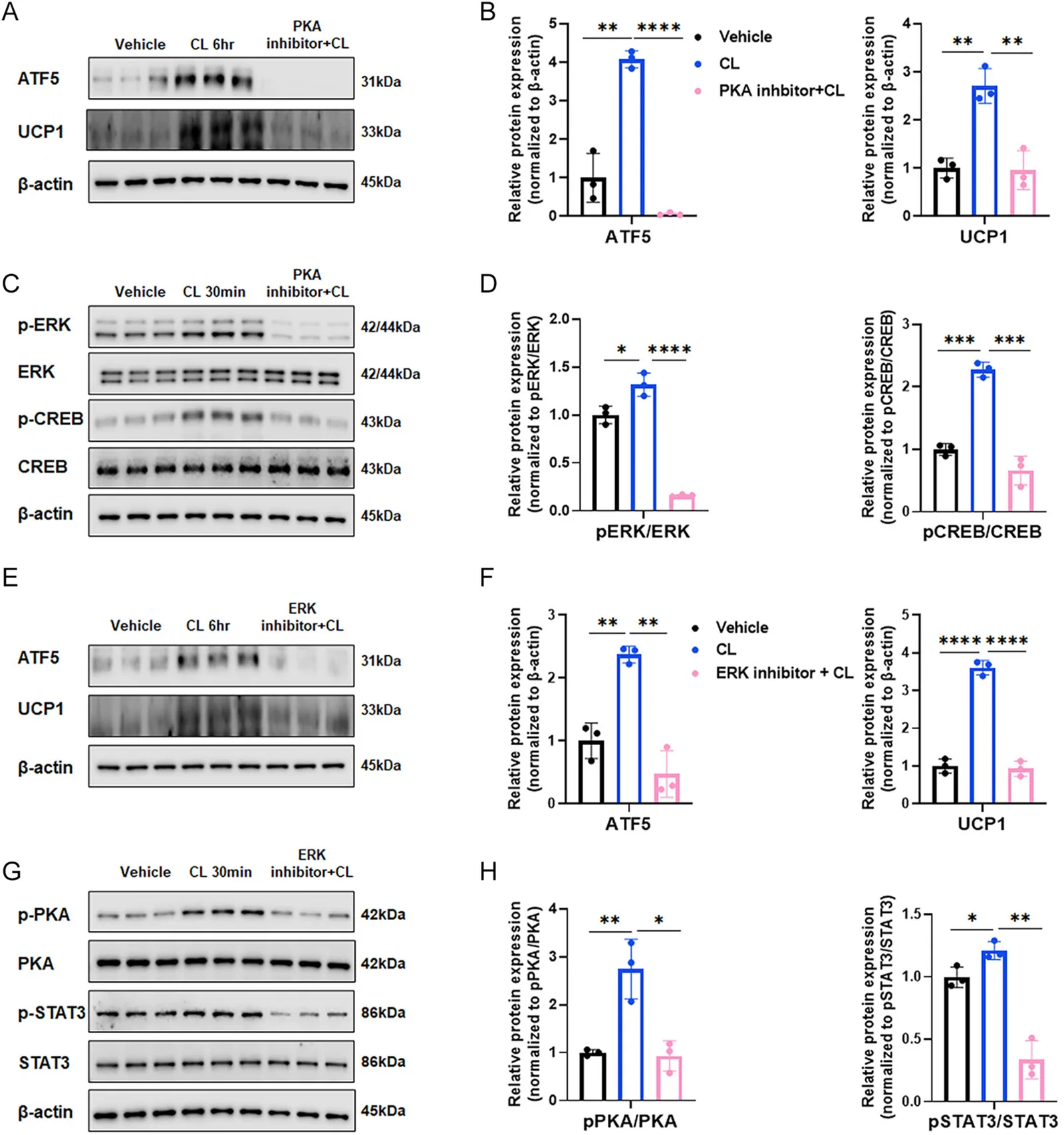
The PKA–ERK signaling axis is required for CL-316,243-induced ATF5 and UCP1 expression in brown adipocytes. (A) Representative immunoblots showing ERK and CREB phosphorylation in differentiated brown adipocytes pretreated with vehicle or the PKA inhibitor KT5720 (1 μM, 1 h), followed by vehicle or CL-316,243 (1 μM, 30 min). β-actin served as a loading control (*n* = 3). (B) Quantification of p-ERK/ERK and p-CREB/CREB levels normalized to vehicle (*n* = 3). (C) Representative immunoblots showing ATF5 and UCP1 protein levels in differentiated brown adipocytes pretreated with vehicle or KT5720 (1 μM, 1 h), followed by vehicle or CL-316,243 (1 μM, 6 h). β-actin served as a loading control (*n* = 3). (D) Quantification of ATF5 and UCP1 protein levels normalized to β-actin and expressed relative to vehicle (*n* = 3). (E) Representative immunoblots showing PKA and STAT3 phosphorylation in differentiated brown adipocytes pretreated with vehicle or the ERK inhibitor LY3214996 (1 μM, 1 h), followed by vehicle or CL-316,243 (1 μM, 30 min). β-actin served as a loading control (*n* = 3). (F) Quantification of p-PKA/PKA and p-STAT3/STAT3 levels normalized to vehicle (*n* = 3). (G) Representative immunoblots showing ATF5 and UCP1 protein levels in differentiated brown adipocytes pretreated with vehicle or LY3214996 (1 μM, 1 h), followed by vehicle or CL-316,243 (1 μM, 6 h). β-actin served as a loading control (*n* = 3). (H) Quantification of ATF5 and UCP1 protein levels normalized to β-actin and expressed relative to vehicle (*n* = 3). Data are presented as mean ± SD from *n* = 3 independent experiments. Statistical significance was determined using one-way ANOVA followed by Dunnett’s multiple comparisons test, as indicated. *P < 0.05, **P < 0.01 versus vehicle. A full-color version of this figure is available at https://doi.org/10.1530/JOE-26-0101.

We next examined whether ERK is similarly required downstream of PKA during CL-316,243 stimulation. Pretreatment with the ERK inhibitor LY3214996 did not alter CL-316,243-induced PKA phosphorylation, confirming its selectivity for ERK signaling, but significantly reduced CL-316,243-induced STAT3 phosphorylation (Fig. 4G and H). Consistent with these findings, LY3214996 pretreatment also suppressed CL-316,243-induced ATF5 and UCP1 protein expression (Fig. 4E and F). Collectively, these results confirm that the PKA–ERK signaling axis identified under basal conditions is directly engaged by β3-adrenergic stimulation and is required for CL-316,243-induced ATF5 and UCP1 expression in brown adipocytes.

### ATF5 sustains UPRmt-associated and lipolytic gene expression, and supports mitochondrial metabolic capacity in differentiated brown adipocytes

To define the functional role of ATF5 in brown adipocytes, *Atf5* expression was silenced using two independent siRNA concentrations (100 and 200 nM) in differentiated cells (Fig. 5A). Quantitative real-time PCR confirmed dose-dependent suppression of *Atf5*, accompanied by a significant reduction in *Ucp1* expression (Fig. 5A and B), indicating that ATF5 contributes to thermogenic gene regulation.

**Figure 5:**
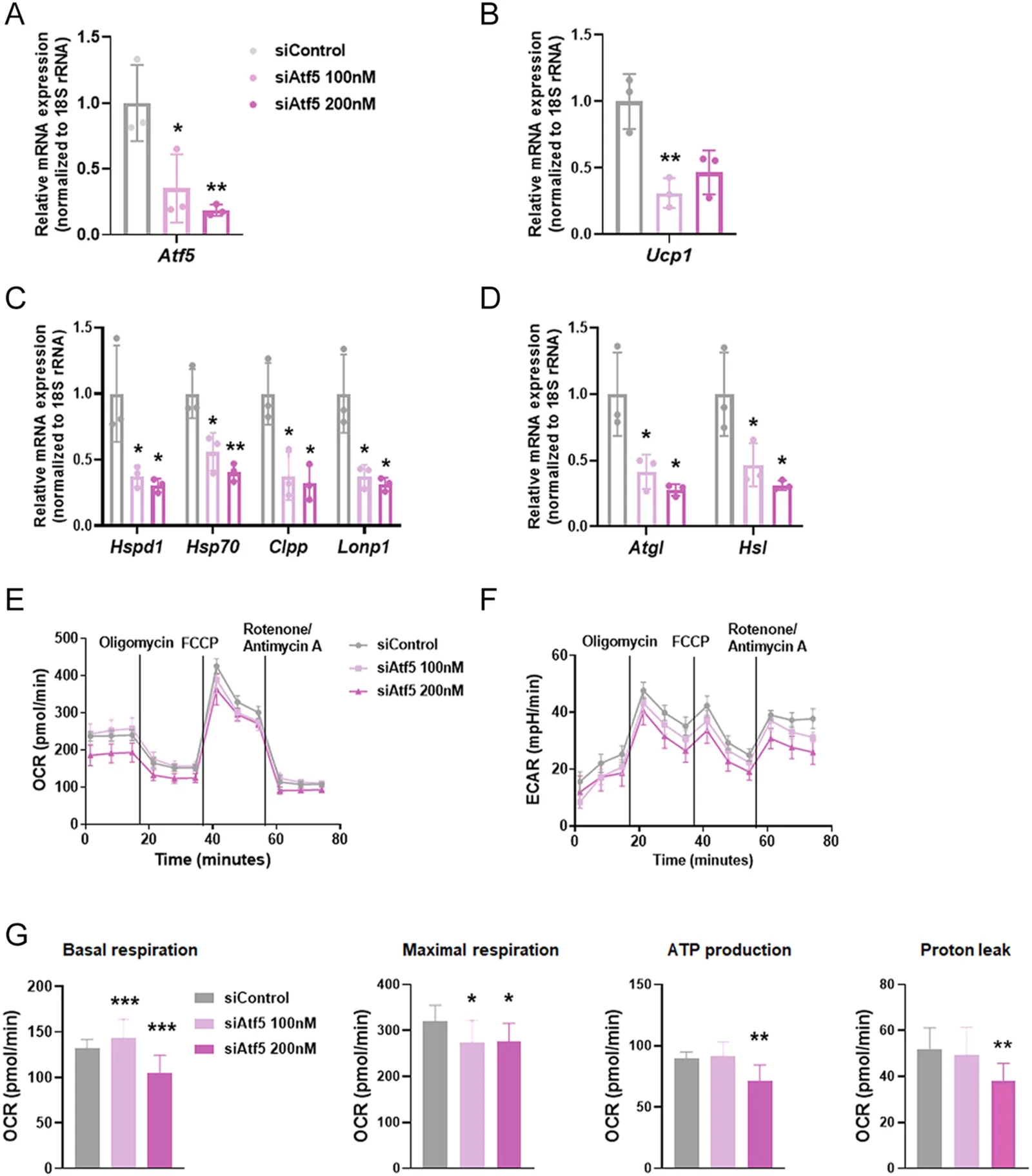
*Atf5* knockdown suppresses UPRmt-related and lipolytic gene expression and impairs mitochondrial respiration in differentiated brown adipocytes *in vitro*. (A–D) Relative mRNA expression of *Atf5* (A), *Ucp1* (B), UPRmt-related genes (*Hspd1, Hspa9, Clpp, and Lonp1*) (C), and lipolytic genes (*Atgl and Hsl*) (D) in differentiated brown adipocytes transfected with siControl or siAtf5 (100 or 200 nM). Gene expression was determined by qRT-PCR, normalized to 18S rRNA, and expressed relative to siControl (*n* = 3). (E and F) Oxygen consumption rate (OCR) (E) and extracellular acidification rate (ECAR) (F) were measured using a Seahorse XF Analyzer. Values were normalized to total cellular protein per well. (G) Quantification of mitochondrial respiration parameters, including basal respiration, maximal respiration, ATP-linked respiration, and proton leak respiration derived from Seahorse analysis. Data are presented as mean ± SD from *n* = 3 independent experiments. Statistical significance was determined using one-way ANOVA followed by Dunnett’s multiple comparisons test, as indicated. *P < 0.05, **P < 0.01 versus siControl. All experiments shown in this figure were performed in cultured differentiated brown adipocytes (*in vitro*). A full-color version of this figure is available at https://doi.org/10.1530/JOE-26-0101.

Given the established role of ATF5 in mitochondrial stress signaling, we next examined the expression of genes related to the mitochondrial unfolded protein response (UPRmt) (9, 10, 11). Silencing of *Atf5* at both concentrations significantly reduced the expression of *Hspd1* (Hsp60), *Hspa9* (Hsp70), *Clpp*, and *Lonp1* (Fig. 5C), suggesting impaired mitochondrial proteostasis. In parallel, expression of key lipolytic genes, including *Atgl* and *Hsl*, was also decreased (Fig. 5D), supporting a role for ATF5 in maintaining lipid mobilization programs.

To assess mitochondrial function, the oxygen consumption rate (OCR) was measured using a Seahorse XF analyzer. *Atf5* knockdown resulted in dose-dependent reductions in basal respiration, maximal respiration, ATP-linked respiration, and proton leak respiration compared with control cells (Fig. 5G), indicating impaired mitochondrial oxidative capacity and reduced UCP1-associated thermogenic function.

We further evaluated glycolytic activity by measuring the extracellular acidification rate (ECAR). Cells with *Atf5* depletion exhibited significantly reduced ECAR responses (Fig. 5F), suggesting that ATF5 loss impairs both oxidative and glycolytic metabolism.

Collectively, these findings demonstrate that ATF5 supports UPRmt-associated and lipolytic gene expression and is required to sustain mitochondrial respiratory and glycolytic capacity in brown adipocytes. The consistent metabolic impairment observed across two independent siRNA doses supports the conclusion that ATF5 plays a central role in maintaining mitochondrial and metabolic homeostasis.

### ATF5 sustains fatty acid oxidation programs and fatty acid–derived carbon flux in brown adipocytes

To determine whether ATF5 regulates fatty acid oxidation (FAO) in brown adipocytes, we first examined the expression of key β-oxidation genes following *Atf5* silencing. Knockdown of *Atf5* significantly reduced the mRNA levels of FAO-associated genes, including *Cpt1a*, *Cpt1b*, and *Acadm* (Fig. 6A), indicating that ATF5 supports transcription of the fatty acid oxidation machinery.

**Figure 6:**
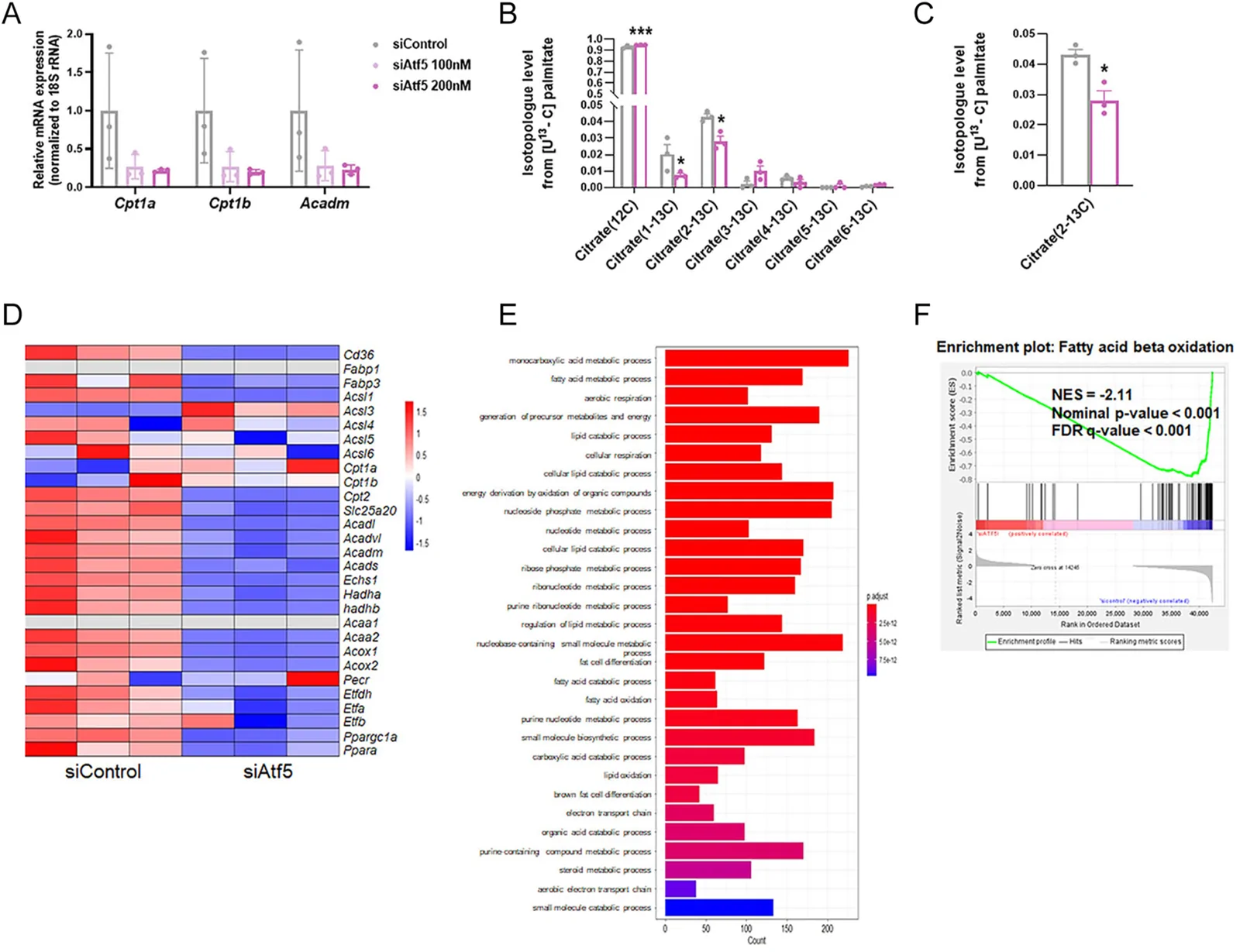
*Atf5* knockdown reduces fatty acid oxidation–related gene expression and limits fatty acid–derived carbon flux into the TCA cycle in differentiated brown adipocytes *in vitro*. (A) Relative mRNA expression of fatty acid oxidation–related genes (*Cpt1a, Cpt1b, and Acadm*) in differentiated brown adipocytes transfected with siControl or siAtf5 (100 or 200 nM). Gene expression was determined by qRT-PCR, normalized to 18S rRNA, and expressed relative to siControl (*n* = 3). (B) Citrate isotopologue distribution derived from [U-^13^C]-palmitate tracing. Isotopologue abundances were normalized to the total citrate pool size. (C) Quantification of M+2 citrate fractional enrichment derived from [U-^13^C]-palmitate (*n* = 3). (D) Heatmap showing relative gene expression (z-score normalized) of genes involved in fatty acid oxidation pathways. (E) Gene ontology (GO) biological process enrichment analysis of differentially expressed genes identified following *Atf5* knockdown. Enriched GO terms are ranked according to gene count, and bar colors indicate adjusted *P* values. (F) Gene set enrichment analysis (GSEA) plot of the fatty acid β-oxidation pathway in siAtf5-treated brown adipocytes. Data are presented as mean ± SD from *n* = 3 independent experiments. Statistical significance was determined using one-way ANOVA followed by Dunnett’s multiple comparisons test, as indicated. *P < 0.05, **P < 0.01 versus siControl. All experiments shown in this figure were performed in cultured differentiated brown adipocytes (*in vitro*). A full-color version of this figure is available at https://doi.org/10.1530/JOE-26-0101.

To assess whether these transcriptional changes translated into altered metabolic flux, we performed [U-^13^C]-palmitate tracing and quantified citrate isotopologue distribution using GC–MS. In control cells, incorporation of palmitate-derived carbons into citrate was readily detected. In contrast, *Atf5* knockdown significantly reduced the fractional enrichment of m+2 citrate (Fig. 6B and C), indicating diminished entry of fatty acid–derived acetyl-CoA into the TCA cycle. These findings demonstrate that loss of ATF5 compromises fatty acid–derived carbon flux into mitochondrial metabolism.

To further define the broader transcriptional landscape associated with ATF5 depletion, we performed transcriptomic analysis in siAtf5-treated brown adipocytes. Heatmap analysis revealed coordinated downregulation of genes involved in fatty acid metabolism and β-oxidation (Fig. 6D). Gene ontology (GO) enrichment analysis further demonstrated significant suppression of biological processes associated with fatty acid metabolism, lipid catabolism, mitochondrial organization, and oxidative metabolism (Fig. 6E). Consistent with these findings, gene set enrichment analysis (GSEA) showed significant negative enrichment of the fatty acid β-oxidation pathway following *Atf5* silencing (Fig. 6F).

To determine whether similar transcriptional alterations were also observed *in vivo*, we analyzed BAT from brown adipocyte-specific *Atf5* knockout mice maintained under room temperature conditions. Gene oncology enrichment analysis revealed suppression of biological processes related to lipid metabolism and mitochondrial function (Supplementary Fig. 3F). Although the *in vivo* RNA-seq analysis was performed using only two biological replicates per group, these transcriptomic findings were consistent with our functional, biochemical, and metabolic analyses and therefore provide supportive evidence for the proposed role of ATF5 in brown adipocyte metabolism.

Collectively, these data indicate that ATF5 is required to sustain fatty acid oxidation–linked transcriptional programs and maintain fatty acid–derived carbon entry into the TCA cycle. Together with the observed defects in mitochondrial respiration, these findings position ATF5 as a regulator coordinating lipid catabolism with mitochondrial metabolic capacity in brown adipocytes.

## Discussion

Brown adipose tissue (BAT) plays a central role in adaptive thermogenesis and systemic energy balance by coordinating lipid mobilization with mitochondrial oxidative metabolism (2, 3, 5, 13, 18). In this study, we identify activating transcription factor 5 (ATF5) as a stress-responsive transcriptional regulator that links β3-adrenergic signaling to mitochondrial metabolic adaptation in brown adipocytes. ATF5 expression was robustly induced by cold exposure and β3-adrenergic stimulation, positioning ATF5 as a downstream effector of adrenergic thermogenic activation.

Mechanistically, our data define a β3-adrenergic–PKA–ERK signaling axis that regulates ATF5 expression. Pharmacological inhibition of PKA or ERK attenuated ATF5 induction and suppressed UCP1 expression under basal conditions, and this suppression was similarly observed when cells were co-stimulated with the β3-adrenergic agonist CL-316,243, confirming that ATF5 induction downstream of adrenergic activation itself depends on PKA–ERK signaling rather than reflecting a nonspecific reduction in basal ATF5/UCP1 turnover (4, 5, 17). Live-cell imaging further demonstrated that β3-adrenergic stimulation selectively enhances nuclear ERK activity, and cold exposure increased nuclear p-ERK abundance in BAT *in vivo*. These findings support a model in which nuclear ERK activation facilitates ATF5-dependent transcriptional responses during thermogenic stimulation (16, 17). While additional studies will be required to determine whether ERK directly modulates ATF5 activity or stability, our data establish nuclear ERK signaling as a key upstream regulator of ATF5 in brown adipocytes.

Beyond its role in thermogenic gene regulation, ATF5 coordinates broader mitochondrial and lipid metabolic programs. Loss of ATF5 reduced the expression of genes involved in fatty acid oxidation (FAO), lipolysis, and the mitochondrial unfolded protein response (UPRmt) and impaired mitochondrial respiration and glycolytic capacity. Stable isotope tracing further demonstrated diminished incorporation of palmitate-derived carbon into the TCA cycle upon ATF5 depletion, indicating compromised fatty acid–derived carbon flux. Transcriptomic and pathway enrichment analyses further supported the coordinated suppression of FAO, oxidative phosphorylation, and mitochondrial metabolic pathways observed *in vitro* and *in vivo* (7, 13, 14, 19). Although the *in vivo* RNA-seq analysis was performed using only two biological replicates per group, the observed transcriptomic changes were consistent with our functional, biochemical, and metabolic analyses, thereby providing supportive evidence for the proposed role of ATF5 in brown adipocyte metabolism (20).

Interestingly, while *Atf5* knockdown efficiency increased in a concentration-dependent manner, the reduction in *Ucp1* expression reached a plateau despite more efficient *Atf5* silencing. This observation suggests that partial suppression of ATF5 may be sufficient to exert maximal effects on *Ucp1* expression under these conditions. Given that *Ucp1* transcription is regulated by multiple signaling pathways and transcriptional regulators, further reduction of ATF5 may not necessarily result in a proportional decrease in *Ucp1* expression.

The observed downregulation of UPRmt-associated genes upon ATF5 depletion indicates that ATF5 contributes to mitochondrial proteostasis under conditions of heightened metabolic demand. This role is consistent with ATF5’s evolutionary conservation as the mammalian ortholog of C. elegans ATFS-1, a regulator of mitochondrial stress signaling (9, 10). However, whether ATF5 directly controls the promoters of thermogenic and FAO-related genes remains to be determined. Chromatin occupancy studies and transcriptional complex mapping will be necessary to define the hierarchical relationship between ATF5 and other members of the ATF/CREB family (10, 21).

ATF5 may function in parallel with or in coordination with ATF4, another stress-responsive transcription factor implicated in mitochondrial remodeling during cold exposure (22). Although both factors are activated under metabolic stress, our data suggest that ATF5 preferentially regulates gene networks enriched for FAO, lipolysis, and UPRmt components. Although multiple members of the ATF family responded to cold exposure (Supplementary Fig. 4E), ATF5 was prioritized in the present study because of its robust induction under thermogenic conditions and its established role in mitochondrial stress adaptation. Moreover, ATF4 protein levels were only modestly affected by inhibition of the PKA–ERK pathway (Supplementary Fig. 4A, B, C, D), suggesting that ATF5 may represent a more prominent downstream effector of β3-adrenergic signaling in brown adipocytes. Nevertheless, the potential contribution of other ATF family members to BAT function warrants further investigation. Future studies employing combinatorial genetic models will be required to delineate the interplay between ATF4 and ATF5 within the integrated stress response in BAT (22). In addition, analysis of a publicly available human brown adipocyte RNA-sequencing dataset (GSE203247) showed that pharmacological activation of lipolysis with SR-3420 was accompanied by increased expression of *ATF5* and *UCP1*, along with genes involved in oxidative phosphorylation and mitochondrial metabolism (Supplementary Fig. 5A and B). Because SR-3420 activates ABHD5/ATGL-mediated lipolysis rather than β3-adrenergic receptor signaling, these data do not directly validate the β3-AR–PKA–ERK–ATF5 pathway in human adipocytes but instead provide supportive evidence linking lipolytic activation with ATF5-associated thermogenic and mitochondrial programs in human brown adipocytes.

Because brown and beige adipocytes share common thermogenic and mitochondrial programs, ATF5 may also contribute to beige adipocyte differentiation and maintenance. Although the present study focused exclusively on classical BAT and differentiated brown adipocytes, the observed effects of ATF5 on the UPRmt, fatty acid oxidation, and mitochondrial respiration suggest that its regulatory functions may extend to beige adipocytes. However, we did not evaluate the effects of *Atf5* deficiency in inguinal or epididymal white adipose tissue, nor did we assess systemic cold sensitivity in *Atf5* knockout mice. Future studies using beige adipocyte models, inducible browning paradigms, and physiological cold challenge experiments will be required to determine whether ATF5 exerts similar roles in beige fat development and whole-body cold adaptation.

PRDM16 is widely recognized as a master regulator of brown adipocyte identity and thermogenic programming (23, 24). In the present study, cold exposure induced a modest increase in *Prdm16* expression; however, this change did not reach statistical significance. In contrast, ATF5 expression was robustly induced under both *in vivo* and *in vitro* thermogenic conditions. To our knowledge, a direct regulatory relationship between PRDM16 and ATF5 has not been established. These observations suggest that ATF5 may primarily regulate mitochondrial metabolic adaptation and stress-responsive programs independently of the core brown adipocyte identity network governed by PRDM16. Further studies will be required to determine whether ATF5 functionally interacts with PRDM16 during thermogenic remodeling.

In summary, our findings identify ATF5 as a transcriptional regulator that integrates β3-adrenergic signaling with mitochondrial stress adaptation and lipid oxidative metabolism. By coordinating UPRmt-associated gene expression, lipolytic activation, and fatty acid–derived carbon entry into the TCA cycle, ATF5 contributes to the maintenance of mitochondrial metabolic capacity required for sustained thermogenic function. These results define a nuclear ERK–ATF5 signaling module that links adrenergic stimulation to mitochondrial metabolic remodeling in brown adipocytes.

## Supplementary materials









## Declaration of interest

The authors declare that there is no conflict of interest that could be perceived as prejudicing the impartiality of this work.

## Funding

This study was supported by the Basic Science Research Program through the National Research Foundation of Korea (NRF), funded by the Ministry of Science, ICT, and Future Planning, Korea (RS-2024-00349240 and NRF-2023R1A2C3006220). Hyon-Seung Yi was supported by a grant from the Korea Health Technology R&D Project through the Korea Health Industry Development Institute (KHIDI), funded by the Ministry of Health & Welfare, Republic of Korea (RS-2022-KH130308). Y K was supported by grants from the Korea Basic Science Institute (National Research Facilities and Equipment Center), the National Research Foundation of Korea (NRF) (RS-2024-00340059 and RS-2024-00398706), the Korea Health Technology R&D Project through the Korea Health Industry Development Institute (KHIDI) (RS-2024-00512909), and the Gwangju Institute of Science and Technology (GIST) research fund (Future-leading Specialized Research Project, 2025). This research was supported by the ANCHOR program through the Daejeon ANCHOR Center, funded by the Ministry of Education (MOE) and the Daejeon Metropolitan City, Republic of Korea (2026-ANCHOR-06-012).

## Author contribution statement

HJJ conceived and designed the study, performed the majority of the experiments, analyzed the data, and drafted the manuscript. YJS performed the nuclear ERK imaging experiments and contributed to imaging data analysis. GML performed the ^13^C-palmitate isotope tracing experiments and analyzed TCA cycle metabolic flux data. JT, HYL, JSM, HTN, TLN, and AMA contributed to experimental assistance and data curation. CK contributed to data curation and validation, and provided scientific advice on experimental design. DWC provided scientific advice and expertise in ^13^C-palmitate isotope tracing and TCA cycle metabolic flux analysis. YHK provided technical guidance and expertise in imaging analysis. HSY provided scientific guidance, supervised the study, secured funding, critically revised the manuscript, and approved the final version for publication. All authors reviewed and approved the final manuscript, agreed to be accountable for the work, and affirmed that all data were generated by the authors. The authorship of this manuscript complies with ethical authorship and publishing guidelines.

## Data availability

The RNA-seq datasets generated in this study have been deposited in the Gene Expression Omnibus (GEO) under accession number GSE315093. All other data supporting the findings of this study are available from the corresponding author upon reasonable request.

## Ethics statement

All animal experiments were conducted in accordance with the guidelines of the Institutional Animal Care and Use Committee (IACUC) and were approved by the IACUC of Chungnam National University Hospital.
