# Supplementary material for: A nuclear ERK–ATF5 axis coordinates mitochondrial metabolism and thermogenic gene expression in brown adipocytes
Source: J Endocrinol. 2026 Oct 19;271(1):e260101. doi: 10.1530/JOE-26-0101 (PMC13695427; doi:10.1530/JOE-26-0101)
Supplement: Supplementary file 1 [file JOE-26-0101_supplementary_figures.pdf]

Supplementary Information

A nuclear ERK–ATF5 axis coordinates mitochondrial metabolism and thermogenic gene expression in brown adipocytes

Short title: Nuclear ERK–ATF5 axis in brown adipocytes

Hyo Ju Jang[1,2†], Yeongjun Suh[3,4†], Jingwen Tian[5], Ho Yeop Lee[1,2], Ji Sun Moon[1], Ha Thi Nga[1,2], Thi Linh Nguyen[1,2], Alfin Mohammad Abdillah[1,2], Gyu Min Lim[7], Dong Wook Choi[7], Chuna Kim[8,9], Yonghoon Kwon[3,4]*, Hyon-Seung Yi[1,2,6]*

[1]Laboratory of Endocrinology and Immune System, Chungnam National University School of Medicine, Daejeon, 35015, Republic of Korea

[2]Department of Medical Science, Chungnam National University School of Medicine, Daejeon, Republic of Korea

[3]Department of Life Sciences, Gwangju Institute of Science and Technology, Gwangju, 61005, Republic of Korea

[4]Integrated Institute of Biomedical Research, Gwangju Institute of Science and Technology, Gwangju, 61005, Republic of Korea

[5]Department of Orthopaedic Surgery, The Sixth Affiliated Hospital of Sun Yat-sen University, Guangzhou, Guangdong 510655, P.R. China

[6]Department of Internal Medicine, Chungnam National University School of Medicine, Daejeon, 35015, Republic of Korea

[7]Department of Biotechnology, College of Life Sciences and Biotechnology, Korea University, Seoul, Republic of Korea

[8]Aging Convergence Research Center, Korea Research Institute of Bioscience and Biotechnology (KRIBB), Daejeon 34141, Republic of Korea

[9]Department of Bioinformatics, KRIBB School of Bioscience, Korea University of Science and Technology, Daejeon 34113, Republic of Korea

[†] These authors contributed equally to this work and share first authorship.

***Corresponding author**

Hyon-Seung Yi, M.D., Ph.D.

Laboratory of Endocrinology and Immune System,

Chungnam National University School of Medicine,

282 Munhwa-ro, Jung-gu, Daejeon 35015, Republic of Korea

Tel: +82-42-280-6994

Email: jmpbooks@cnu.ac.kr

Yonghoon Kwon, Ph.D.

Department of Life Sciences,

Gwangju Institute of Science and Technology,

Gwangju, 61005, Republic of Korea

Email: yonghoon@gist.ac.kr

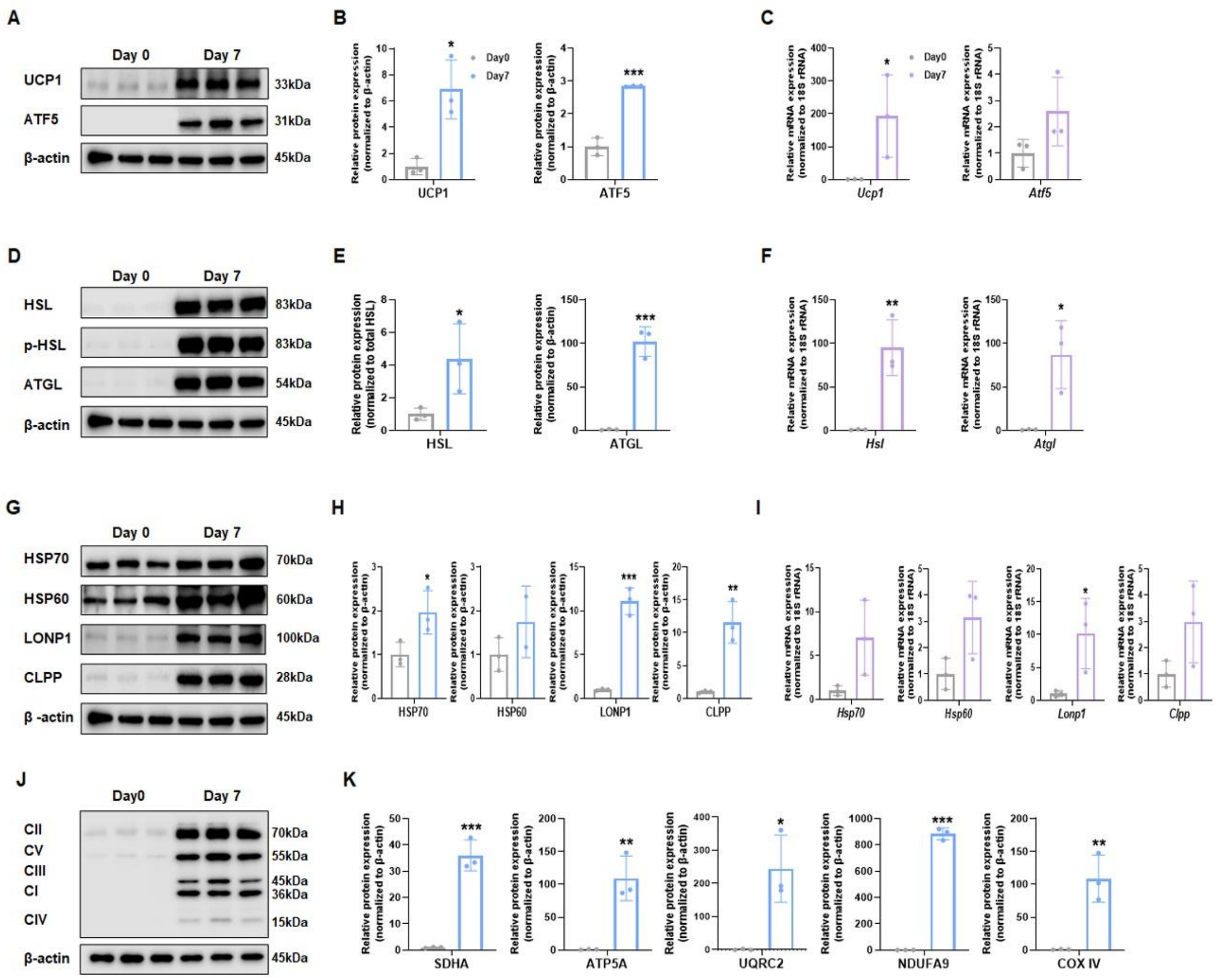

Supplementary figure 1. Brown adipocyte differentiation enhances thermogenic, lipolytic, mitochondrial stress–response, and oxidative phosphorylation programs.

(A) Immunoblot analysis of UCP1 and ATF5 in brown adipocytes at Day 0 and Day 7 of differentiation. β-Actin served as a loading control. (B) Quantification of UCP1 and ATF5 protein expression normalized to β-actin. (C) Relative mRNA expression of *Ucp1* and *Atf5* at Day 0 and Day 7. (D) Immunoblot analysis of lipolytic proteins (phospho-HSL, total HSL, ATGL). (E) Quantification of phospho-HSL/HSL ratios and ATGL protein levels. (F) Relative mRNA expression of *Hsl* and *Atgl*. (G) Immunoblot analysis of mitochondrial stress–response proteins (HSP70, HSP60, LONP1, CLPP). (H) Quantification of corresponding protein levels. (I) Relative mRNA expression of UPRmt-related genes (*Hsp70*, *Hsp60*, *Lonp1*, *Clpp*). (J) Immunoblot analysis of mitochondrial oxidative phosphorylation

complexes (CI–CV). (K) Quantification of representative OXPHOS subunits (SDHA, ATP5A, UQCRC2, NDUFA9, COX IV). Data are presented as mean ± SD from three independent experiments (n = 3). Statistical significance was determined using an unpaired two-tailed Student's t-test. *P < 0.05, **P < 0.01, ***P < 0.001 versus Day 0.

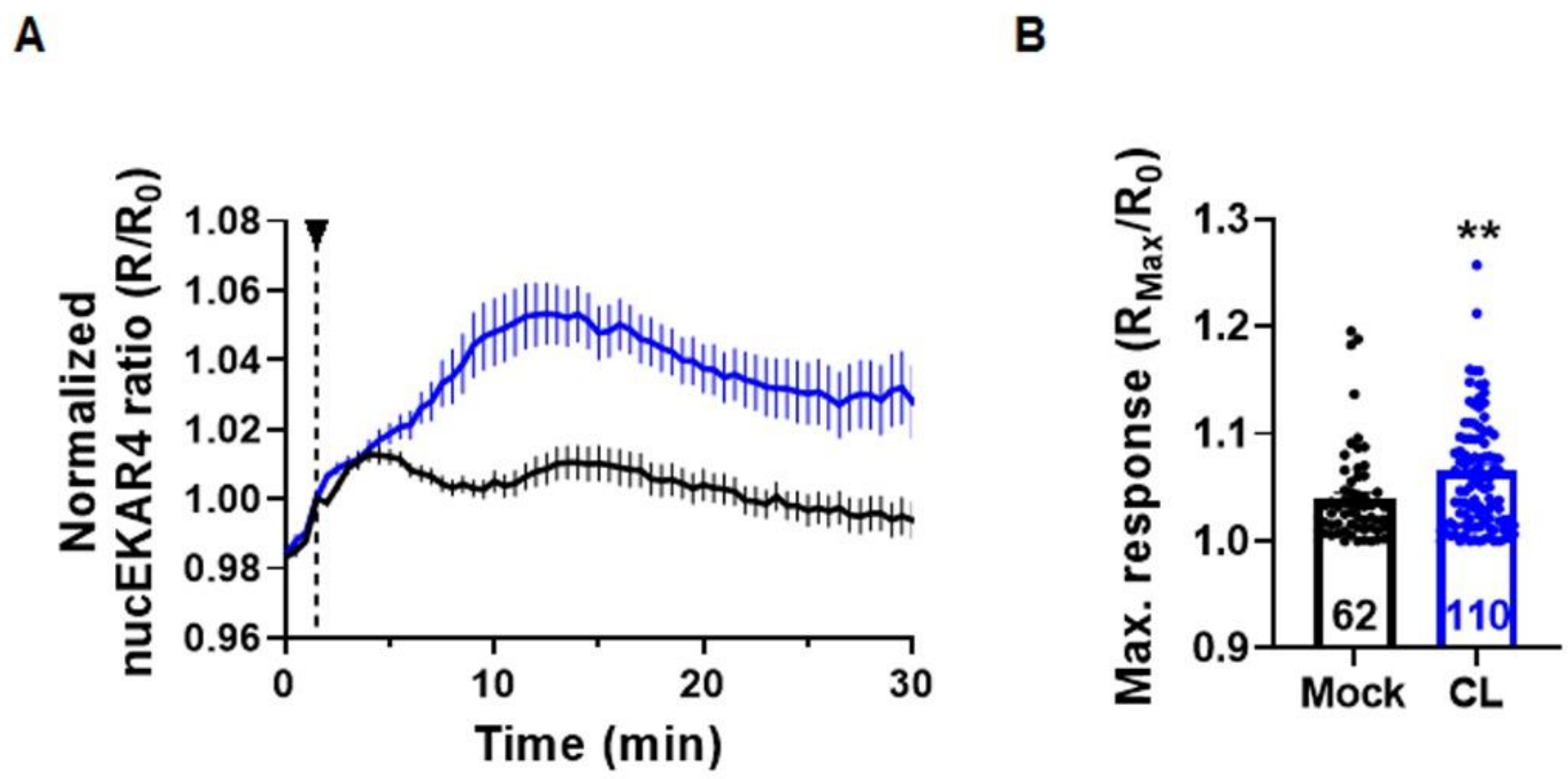

Supplementary figure 2. β3-adrenergic stimulation activates nuclear ERK signaling in undifferentiated brown adipocytes.

(A) Representative time-course traces of normalized Y/C emission ratio (R/R_0) from undifferentiated brown adipocytes expressing the nuclear ERK biosensor nucEKAR4. Cells were treated with vehicle (Mock; black) or CL-316,243 (blue), and fluorescence emission ratios were monitored over time. CL-316,243 stimulation induced a rapid increase in nuclear ERK activity compared with vehicle-treated controls. (B) Quantification of maximal nucEKAR4 responses ($Rmax/R_0$). Each dot represents a single cell (n = 62 [Mock]; n = 110 [CL-316,243]) pooled from three independent experiments. Data are presented as mean ± SEM. Statistical significance was determined using an unpaired two-tailed Student's t-test. p = 0.0002 versus Mock.

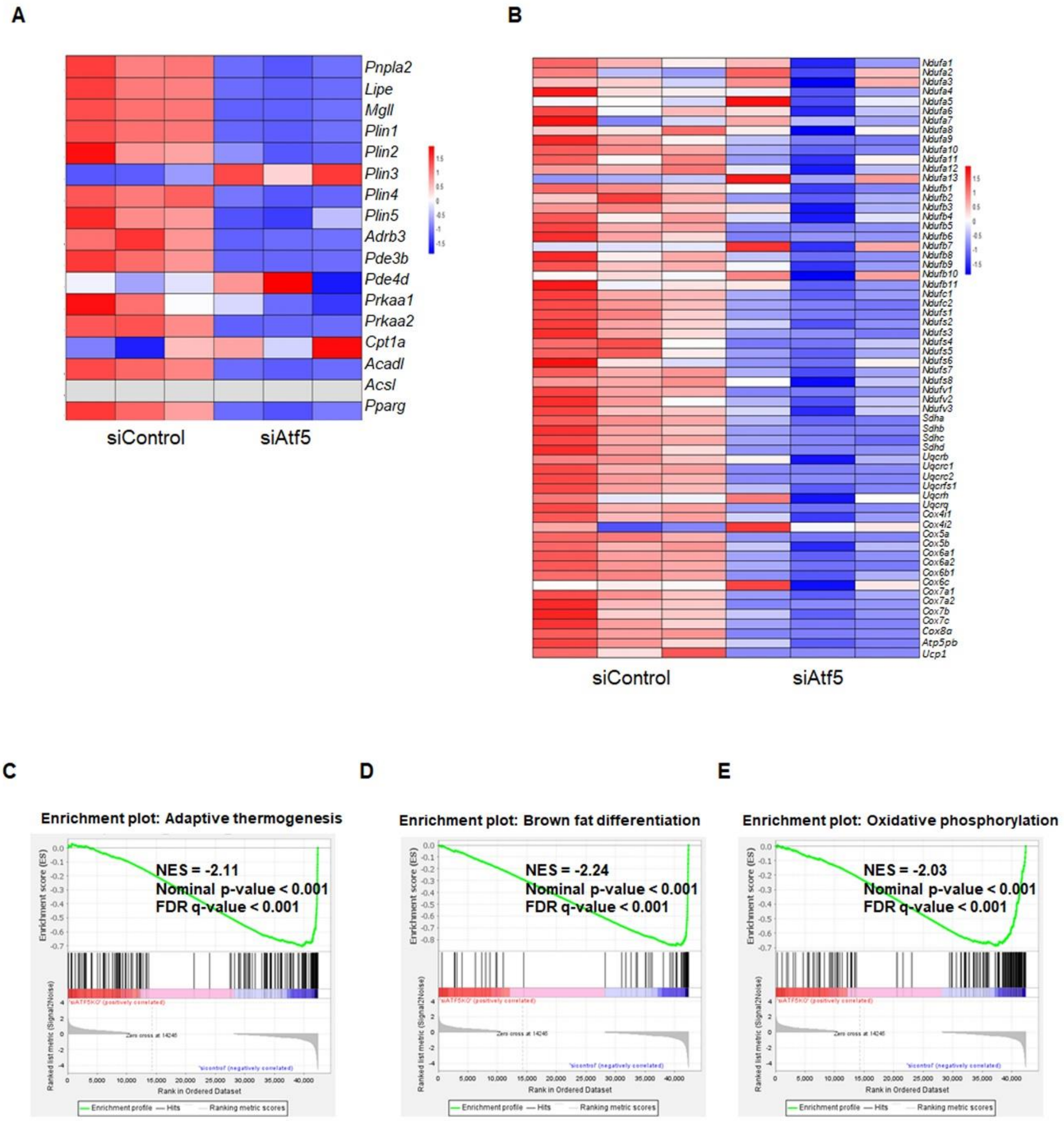

A
Pnpla2
Lipe
Mgll
Plin1
Plin2
Plin3
Plin4
Plin5
Adrb3
Pde3b
Pde4d
Prkaa1
Prkaa2
Cpt1a
Acadl
Acsl
Pparg
siControl
siAtf5
B
Ndufa1
Ndufa2
Ndufa3
Ndufa4
Ndufa5
Ndufa6
Ndufa7
Ndufa8
Ndufa9
Ndufa10
Ndufa11
Ndufa12
Ndufa13
Ndufb1
Ndufb2
Ndufb3
Ndufb4
Ndufb5
Ndufb6
Ndufb7
Ndufb8
Ndufb9
Ndufb10
Ndufb11
Ndufc1
Ndufc2
Ndufs1
Ndufs2
Ndufs3
Ndufs4
Ndufs5
Ndufs6
Ndufs7
Ndufs8
Ndufv1
Ndufv2
Ndufv3
Sdha
Sdhb
Sdhc
Sdhd
Uqcrb
Uqcrc1
Uqcrc2
Uqcrfs1
Uqcrh
Uqcrq
Cox4i1
Cox4i2
Cox5a
Cox5b
Cox6a1
Cox6a2
Cox6b1
Cox6c
Cox7a1
Cox7a2
Cox7b
Cox7c
Cox8a
Atp5pb
Ucp1
siControl
siAtf5
C
Enrichment plot: Adaptive thermogenesis
NES = -2.11
Nominal p-value < 0.001
FDR q-value < 0.001
D
Enrichment plot: Brown fat differentiation
NES = -2.24
Nominal p-value < 0.001
FDR q-value < 0.001
E
Enrichment plot: Oxidative phosphorylation
NES = -2.03
Nominal p-value < 0.001
FDR q-value < 0.001

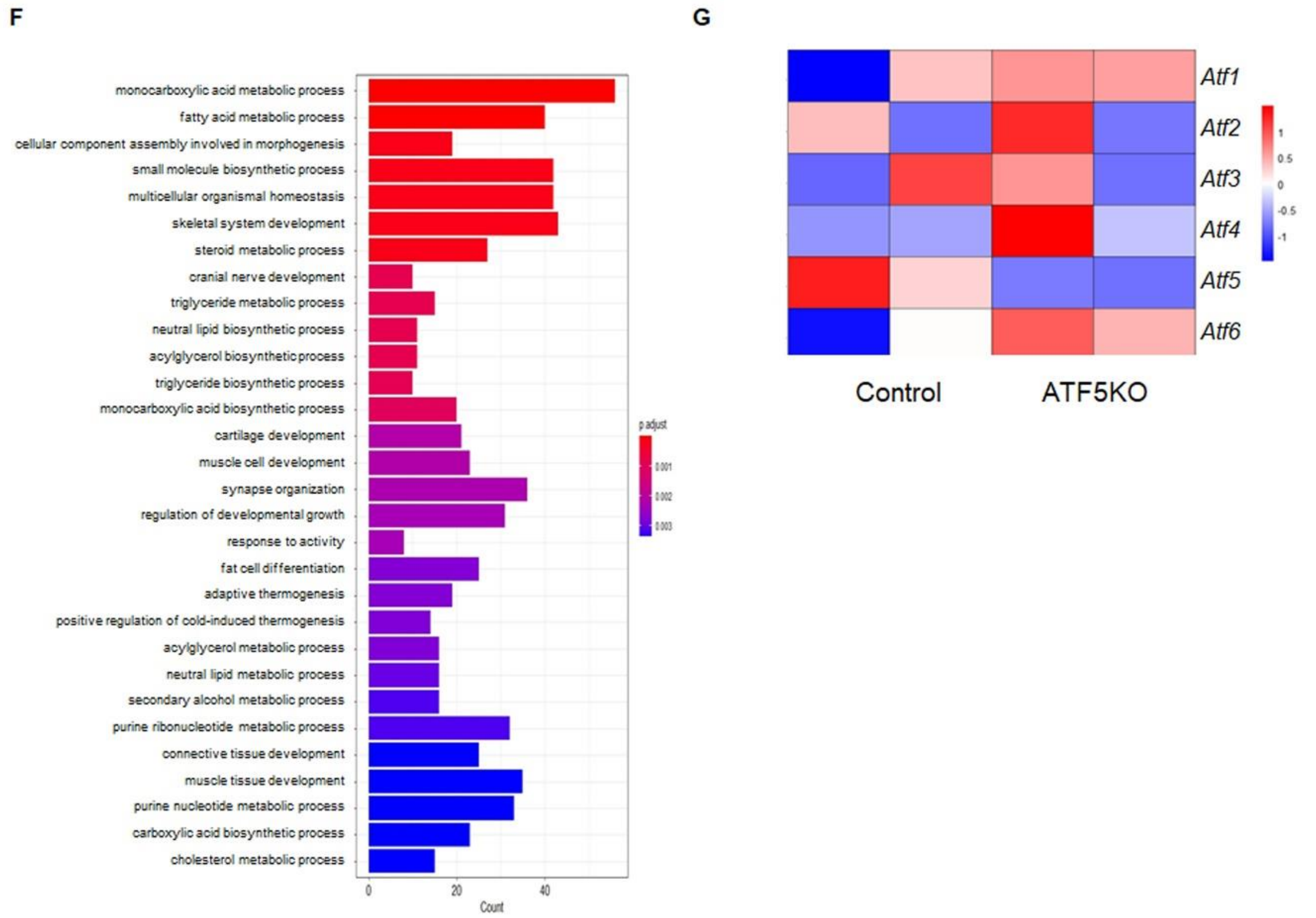

Supplementary figure 3. Transcriptomic and pathway analyses of Atf5-silenced brown adipocytes (*in vitro*) and ATF5-deficient BAT (in vivo). (A–C, E–G) Analyses performed using differentiated brown adipocytes transfected with siAtf5 (*in vitro*).

(A) Heatmap showing representative metabolic and thermogenic genes in siControl vs siAtf5-treated differentiated brown adipocytes (in vitro, n = 3 biological replicates per group). (B) Heatmap showing genes involved in oxidative phosphorylation and mitochondrial electron transport chain complexes. (C–E) Gene set enrichment analysis (GSEA) plots showing enrichment of adaptive thermogenesis (C), brown adipocyte differentiation (D), and oxidative phosphorylation (E) pathways in siAtf5-treated differentiated brown adipocytes. (F) GO enrichment analysis of differentially expressed genes. (G) Heatmap showing expression of ATF family members in BAT from brown adipocyte-specific ATF5-deficient mice (Ucp1-Cre; Atf5^fl/fl^) and littermate controls maintained under basal conditions (in vivo, n = 2

biological replicates per group).

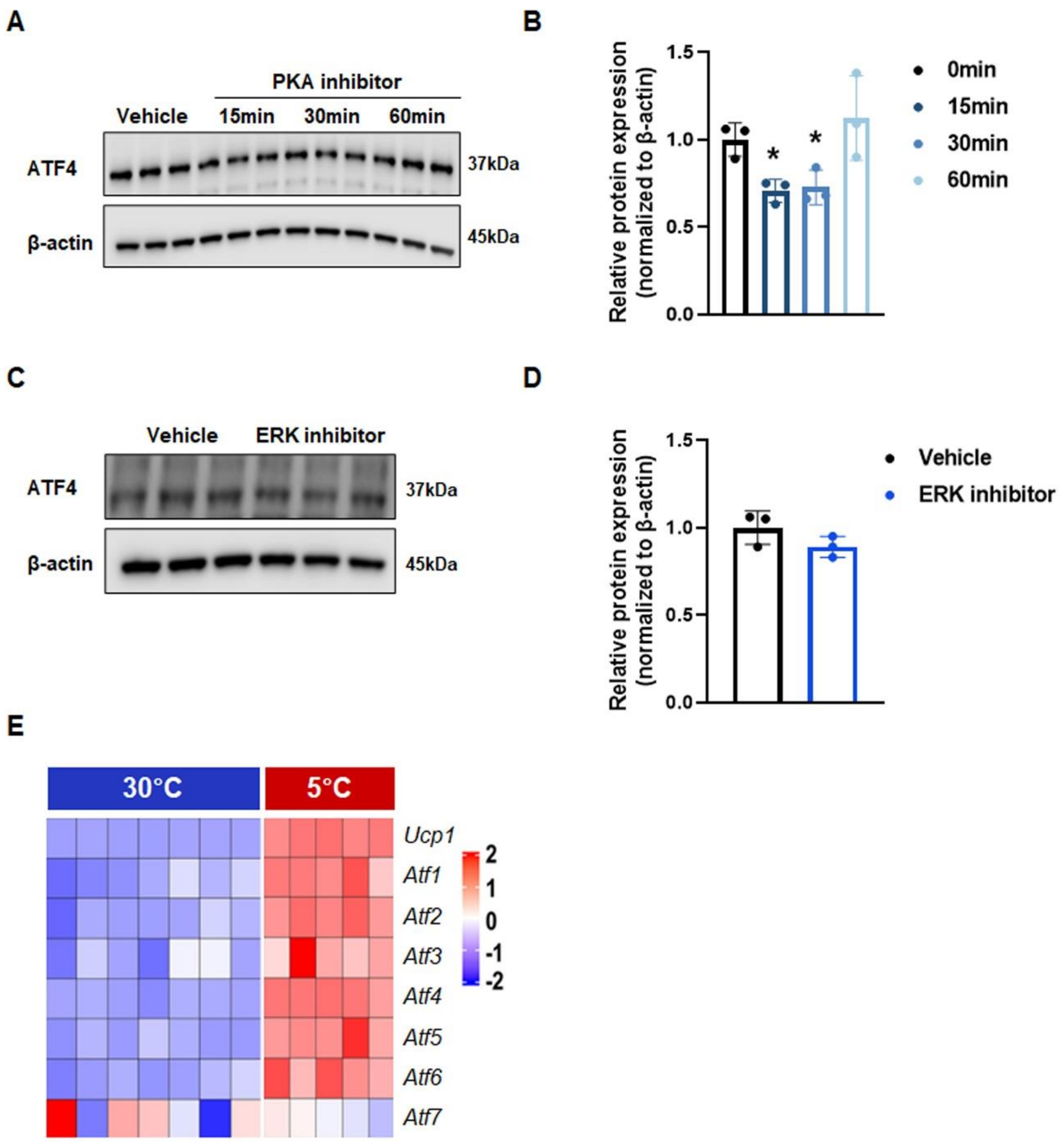

Supplementary figure 4. Additional analyses supporting the selection of ATF5 as the primary target transcription factor.

(A) Representative immunoblot of ATF4 protein levels in differentiated brown adipocytes treated with the PKA inhibitor KT5720 for the indicated times. (B) Quantification of ATF4 protein levels normalized to β-actin. Data are presented as mean ± SD from three independent

experiments. *P < 0.05 versus 0 min. (C) Representative immunoblot showing ATF4 protein levels following ERK inhibition. (D) Quantification of ATF4 protein levels normalized to β-actin. Data are presented as mean ± SD from three independent experiments; no significant difference was observed between vehicle and ERK inhibitor groups. (E) Heatmap showing expression of Atf family members in BAT from mice housed at 30°C or 5°C (GSE147392).

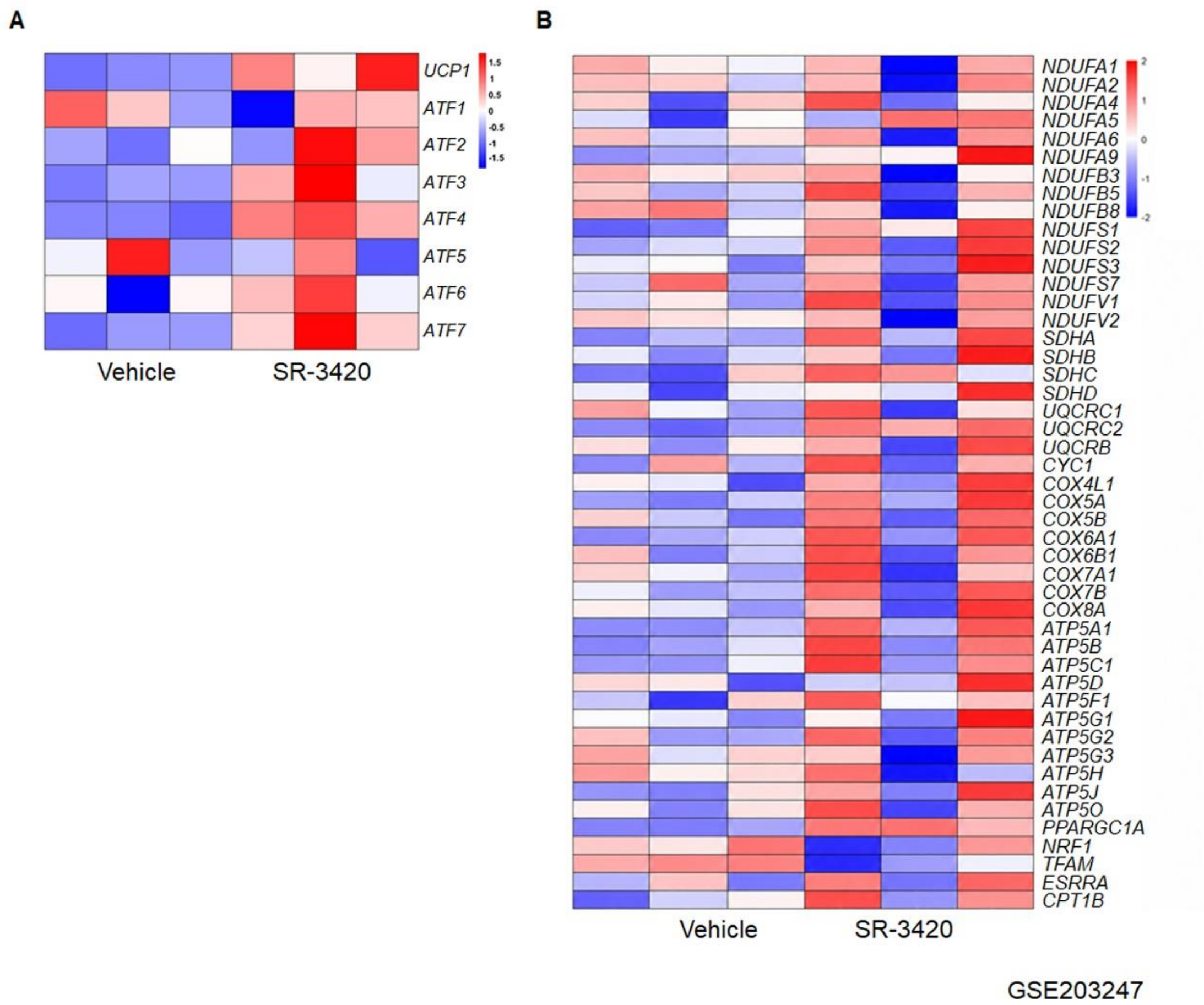

Supplementary figure 5. Human adipocyte transcriptomic analysis supports an association between ATF5 and thermogenic metabolic programs.

(A) Heatmap showing the expression patterns of *UCP1* and *ATF* family members (*ATF1–ATF7*) in vehicle- and SR-3420-treated human brown adipocytes using the publicly available RNA-sequencing dataset GSE203247. (B) Heatmap showing the expression of oxidative phosphorylation- and mitochondrial metabolism-related genes, including electron transport

chain components and thermogenic regulators (*PPARGC1A*, *NRF1*, *TFAM*, *ESRRA*, *and CPT1B*), in vehicle- and SR-3420-treated human brown adipocytes (GSE203247).
