# Supplementary material for: A nuclear ERK–ATF5 axis coordinates mitochondrial metabolism and thermogenic gene expression in brown adipocytes
Source: J Endocrinol. 2026 Oct 19;271(1):e260101. doi: 10.1530/JOE-26-0101 (PMC13695427; doi:10.1530/JOE-26-0101)
Supplement: Supplementary file 2 [file JOE-26-0101_supplementary_tables.pdf]

Supplementary tables

A nuclear ERK–ATF5 axis coordinates mitochondrial metabolism and thermogenic gene expression in brown adipocytes

Short title: Nuclear ERK–ATF5 axis in brown adipocytes

Hyo Ju Jang[1,2†], Yeongjun Suh[3,4†], Jingwen Tian[5], Ho Yeop Lee[1,2], Ji Sun Moon[1], Ha Thi Nga[1,2], Thi Linh Nguyen[1,2], Alfin Mohammad Abdillah[1,2], Gyu Min Lim[7], Dong Wook Choi[7], Chuna Kim[8,9], Yonghoon Kwon[3,4]*, Hyon-Seung Yi[1,2,6]*

[1]Laboratory of Endocrinology and Immune System, Chungnam National University School of Medicine, Daejeon, 35015, Republic of Korea

[2]Department of Medical Science, Chungnam National University School of Medicine, Daejeon, Republic of Korea

[3]Department of Life Sciences, Gwangju Institute of Science and Technology, Gwangju, 61005, Republic of Korea

[4]Integrated Institute of Biomedical Research, Gwangju Institute of Science and Technology, Gwangju, 61005, Republic of Korea

[5]Department of Orthopaedic Surgery, The Sixth Affiliated Hospital of Sun Yat-sen University, Guangzhou, Guangdong 510655, P.R. China

[6]Department of Internal Medicine, Chungnam National University School of Medicine, Daejeon, 35015, Republic of Korea

[7]Department of Biotechnology, College of Life Sciences and Biotechnology, Korea University, Seoul, Republic of Korea

[8]Aging Convergence Research Center, Korea Research Institute of Bioscience and

Biotechnology (KRIBB), Daejeon 34141, Republic of Korea

[9]Department of Bioinformatics, KRIBB School of Bioscience, Korea University of Science and Technology, Daejeon 34113, Republic of Korea

† These authors contributed equally to this work and share first authorship.

***Corresponding author**

Hyon-Seung Yi, M.D., Ph.D.

Laboratory of Endocrinology and Immune System,

Chungnam National University School of Medicine,

282 Munhwa-ro, Jung-gu, Daejeon 35015, Republic of Korea

Tel: +82-42-280-6994

Email: jmpbooks@cnu.ac.kr

Yonghoon Kwon, Ph.D.

Department of Life Sciences,

Gwangju Institute of Science and Technology,

Gwangju, 61005, Republic of Korea

Email: yonghoon@gist.ac.kr

Table 1. List of primer sequences

primer	Forward (5'→3')	Reverse (5'→3')
18s	CTG GTT GAT CCT GCC AGT AG	CGA CCA AAG GAA CCA TAA CT
Cpt1a	TAT AAC AGG TGG TTT GAC	CAG AGG TGC CCA ATG ATG
Cpt1b	TCG CAG GAG AAA ACA CCA TGT	AAC AGT GCT TGG CGG ATG TG
Acadm	TGA CGG AGC AGC CAA TGA	TCG TCA CCC TTC TTC TCT GCT T
Atf5	TGG GCT GGC TCG TAG ACT AT	GTC ATC CAA TCA GAG AAG CCG
Ucp1	CAC CTT CCC GCT GGA CAC T	CCC TAG GAC ACC TTT ATA CCT AAT GG
Dio2	CAG TGT GGT GCA CTG CTC CAA TC	TGA ACC AAA GTT GAC CAC CAG
Prdm16	CAC ACG GAA GAG CGT GAG TA	CCG TGA ACA CCT TGA CAC AG
Hsl	AGA CAC CAG CCA ACG GAT AC	GCG GTT AGA AGC CAC ATA GC
Atgl	ACT GTG GCC TCA TTC CTC CT	AAC TGG ATG CTG GTG TTG GT
Hspa9	CAA GGA TGC CCA AGG	TAA CGT CAC CAG CCA

	TTC AG	ACA CA
Hspd1	GAG CTG GGT CCC TCA CTC G	AGT CGA AGC ATT TCT GCG GG
Lonp1	AGC CCT ATG TTG GCG TCT TC	CCG GCT GAT GTG AAT CCT TCT
Clpp	GCC ATT CAC TGC CCA ATT CC	TGC TGA CTC GAT CAC CTG TAG
Pgc1α	TCT CAG TAA GGG GCT GGT TG	AGC AGC ACA CTC TAT GTC ACT C

Table 2. List of antibodies

Antibody	Company	Catalog #
UCP1	Abcam	ab10983
Total OXPHOS (cocktail)	Abcam	ab110412
ATF5	Abcam	ab184923
HSL	Cell Signaling	4107S
p-HSL	Cell Signaling	45804
ATGL	Cell Signaling	2138S
β-Actin	Cell Signaling	4967S
HSP70	Invitrogen	MA3-006
HSP60	Cell Signaling	12165S
LONP1	Proteintech	66043-1-Ig
CLPP	Invitrogen	PA5-52722
ERK	Cell Signaling	9102S
p-ERK	Cell Signaling	4376S
PKA	Cell Signaling	4782S
Phospho-PKA	Cell Signaling	4781S
CREB	Cell Signaling	9197S
Phospho-CREB	Cell Signaling	9198S
STAT3	Cell Signaling	9139S
p-STAT3	Cell Signaling	9145S
GAPDH	Cell Signaling	5174S
LaminB1	Santa cruz	SC-374015

ATF4	Abcam	ab31390
